# A Mutant RNA Polymerase Activates the General Stress Response, Enabling Escherichia coli Adaptation to Late Prolonged Stationary Phase

**DOI:** 10.1128/mSphere.00092-20

**Published:** 2020-04-15

**Authors:** Pabitra Nandy, Savita Chib, Aswin Seshasayee

**Affiliations:** a National Centre for Biological Sciences, Bangalore, India; University of Iowa

**Keywords:** *E. coli*, GASP, RNA polymerase, stationary phase, stress response

## Abstract

Escherichia coli populations undergo repeated replacement of parental genotypes with fitter variants deep in stationary phase. We isolated one such variant, which emerged after 3 weeks of maintaining an E. coli K-12 population in stationary phase. This variant displayed a small colony phenotype and slow growth and was able to outcompete its ancestor over a narrow time window in stationary phase. The variant also shows tolerance to beta-lactam antibiotics, though not previously exposed to the antibiotic. We show that an RpoC(A494V) mutation confers the slow growth and small colony phenotype on this variant. The ability of this mutation to confer a growth advantage in stationary phase depends on the availability of the stationary-phase sigma factor σ^S^. The RpoC(A494V) mutation upregulates the σ^S^ regulon. As shown over 20 years ago, early in prolonged stationary phase, σ^S^ attenuation, but not complete loss of activity, confers a fitness advantage. Our study shows that later mutations enhance σ^S^ activity, either by mutating the gene for σ^S^ directly or via mutations such as RpoC(A494V). The balance between the activities of the housekeeping major sigma factor and σ^S^ sets up a trade-off between growth and stress tolerance, which is tuned repeatedly during prolonged stationary phase.

**IMPORTANCE** An important general mechanism of a bacterium’s adaptation to its environment involves adjusting the balance between growing fast and tolerating stresses. One paradigm where this plays out is in prolonged stationary phase: early studies showed that attenuation, but not complete elimination, of the general stress response enables early adaptation of the bacterium E. coli to the conditions established about 10 days into stationary phase. We show here that this balance is not static and that it is tilted back in favor of the general stress response about 2 weeks later. This can be established by direct mutations in the master regulator of the general stress response or by mutations in the core RNA polymerase enzyme itself. These conditions can support the development of antibiotic tolerance although the bacterium is not exposed to the antibiotic. Further exploration of the growth-stress balance over the course of stationary phase will necessarily require a deeper understanding of the events in the extracellular milieu.

## Abstract (precis)

An important general mechanism of a bacterium’s adaptation to its environment involves adjusting the balance between growing fast and tolerating stresses. One paradigm where this plays out is in prolonged stationary phase: early studies showed that attenuation, but not complete elimination, of the general stress response enables early adaptation of the bacterium E. coli to the conditions established about 10 days into stationary phase. We show here that this balance is not static and that it is tilted back in favor of the general stress response about 2 weeks later. This can be established by direct mutations in the master regulator of the general stress response or by mutations in the core RNA polymerase enzyme itself. These conditions can support the development of antibiotic tolerance although the bacterium is not exposed to the antibiotic. Further exploration of the growth-stress balance over the course of stationary phase will necessarily require a deeper understanding of the events in the extracellular milieu.

## INTRODUCTION

Under standard laboratory conditions, well-aerated cultures of Escherichia coli exhibit three distinct phases of growth within 24 h of inoculation: the initial slow-growing lag phase, followed by a period of exponential growth and finally the stationary phase during which no appreciable change in cell number is observed. Most of the cells in culture lose viability within 2 to 5 days after reaching stationary phase (1, 2). The small surviving subpopulation can remain viable for at least 60 months in a phase known as “prolonged stationary phase” or “long-term stationary phase” (LTSP) (1, 3, 4). Prolonged stationary phase might represent a laboratory approximation of different natural habitats populated by bacteria, many of which are nutrient limited (2).

Although the total number of viable cells does not appreciably change in this phase, prolonged stationary phase is dynamic and is characterized by repeated replacement of parental genotypes (and phenotypes) by a different set of genetic variants better adapted to the ambient environment (2). Populations from aged cultures outcompete younger cultures in prolonged stationary phase, a phenotype known as growth advantage in stationary phase (GASP) (4). In this phase, both genetic and phenotypic diversity increase over time (2, 5).

Mutations in different global regulators of gene expression confer GASP on their host cells. The most prominent among these are in the sigma factor σ^S^, the master activator of the general stress response. An elongated and attenuated variant of σ^S^, named *rpoS819*, was the first gene to be implicated in GASP (4). Other alleles conferring GASP include mutations in Lrp, a global regulator controlling amino acid metabolism (6); activation of the *bgl* operon (7) by mechanisms that attenuate H-NS, the global regulator responsible for transcriptional repression of horizontally acquired genes (8); and mutations in CpdA, the cyclic AMP phosphodiesterase (9), which might influence the hierarchy of carbon source utilization. Several mutations in the core RNA polymerase subunits have been observed in deep-sequencing-based studies of the genotype space explored in prolonged stationary phase (5, 10, 11). Previous research has described the emergence of diverse colony morphologies in prolonged stationary phase (3). Consistent with these observations, we isolated a variant characterized by its small colony size growing alongside cells forming “normal”-size colonies, after maintaining E. coli in stationary phase for over 3 weeks.

Slow-growing mutants of various bacteria can withstand a variety of stresses targeting growth-associated processes. For example, slow-growing E. coli variants deficient in the electron transport chain can tolerate aminoglycosides whose transport into the cells is an active process (12). A certain class of slow-growing bacteria, called “small colony variants” (SCV) emerge in response to stress in the environment (13) and have been discovered in pathogenic contexts. These variants arise from metabolic deficiencies, particularly in thymidine biosynthesis or in the electron transport chain. Some of these variants are known to evade host defense systems and the action of various antibiotics, while forming persister populations responsible for recalcitrant diseases (14).

In this study, we describe the phenotypic characteristics and genetic underpinnings of a slow-growing, small-colony-forming variant of Escherichia coli isolated 3 weeks into stationary phase in nutrient-rich medium (LB) and show that a mutation in the core RNA polymerase can confer GASP on its bearer by activating the σ^S^ regulon.

## RESULTS

### Small colony phenotype evolved from ZK819 in prolonged stationary phase.

**FIG 1:**
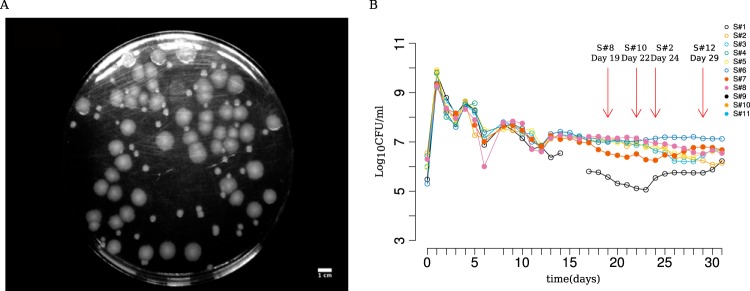
Evolving a GASP-enabled strain (ZK819) in prolonged stationary phase and the emergence of small colony forms. (A) Representative picture of small colonies as seen on an LB agar plate spread with a 29-day-old culture of ZK819. (B) Daily colony count of 11 independent replicates of E. coli ZK819 for 30 days in prolonged stationary phase. First recorded emergences of small colonies in different replicate lines are marked by red arrows.

In a previous study (5), we had propagated 4 replicate populations of Escherichia coli ZK819 in rich medium (LB) for 30 days without the addition of external nutrients after inoculation. ZK819 is the pioneer GASP strain of E. coli, carrying *rpoS819*, an elongated and attenuated variant of the sigma factor for the general stress response σ^S^. We repeated the long-term evolution experiment in LTSP with 11 replicates and followed the cultures daily for 30 days (Fig. 1B). In this experiment, colonies with different morphologies emerged over time: mucoid colonies, followed by fragmented colonies and small colonies. Variants with small colony (SC) phenotype first appeared on day 19 and remained for as long as the cultures were examined (Fig. 1) (5). We isolated an SC from these long-term stationary-phase experiments for further study and describe its characteristics here.

10.1128/mSphere.00092-20.4 TABLE S1 Primers used in this study. Download Table S1, PDF file, 0.02 MB. Copyright © 2020 Nandy et al. 2020 Nandy et al. This content is distributed under the terms of the Creative Commons Attribution 4.0 International license.

We briefly note here that the appearance of the SC phenotype is repeatable, and we observed its appearance, after 19 to 22 days postinoculation, in 4 out of 11 additional prolonged stationary-phase runs (see Table S2 in the supplemental material). These were not studied in greater detail here.

10.1128/mSphere.00092-20.5 TABLE S2 List of unique mutations identified in different replicate lines of ZK819 which gave rise to SCV in the second run of evolution experiment in prolonged stationary phase. Unique mutations were determined by the presence of the mutation at 100% frequency in the population as well as absence from coexisting strain LCV and ancestor strain ZK819. Download Table S2, PDF file, 0.04 MB. Copyright © 2020 Nandy et al. 2020 Nandy et al. This content is distributed under the terms of the Creative Commons Attribution 4.0 International license.

### SC exhibits slow growth and increased tolerance to beta-lactam antibiotics compared to ancestor and coexisting strains.

**FIG 2:**
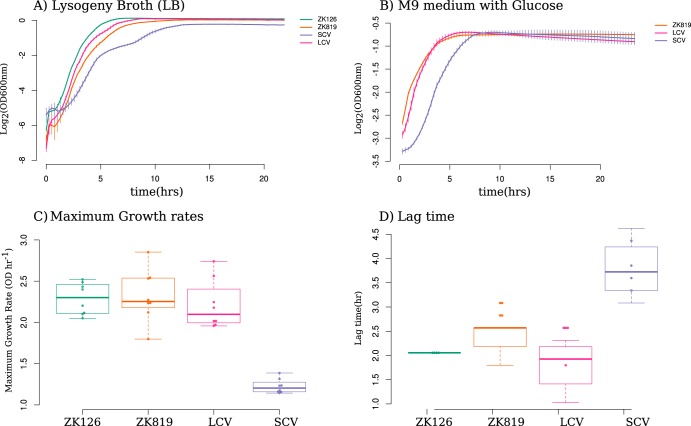
Growth characteristics of SC. (A and B) Growth curves of SC compared to coexisting strain (LCV), evolutionary ancestor (ZK819), and parent of ancestor (ZK126) in rich (LB) (A) and minimal (M9 plus glucose) (B) media. (C) Distribution of maximum growth rates of SCV, LCV, and ancestor strains. Error bars indicate standard deviations (*n* = 8). (D) Distribution of lag time for SCV, LCV, and ancestor strains. Error bars indicate standard deviations (*n* = 8).

We measured the growth of SC, a coexisting variant with ancestral colony size (LC), ZK819 (the strain which was used to initiate our GASP experiment), and ZK126 (the parent of ZK819, used by the Kolter lab in reference 3) in LB (Fig. 2A). SC exhibited slow growth, in terms of both maximum growth rate (Fig. 2C) and lag times compared to ZK819 and LC (Fig. 2D). All three strains showed similar stationary-phase yields in rich media. In minimal medium supplemented with glucose as sole carbon source, SC retained its slow growth (Fig. 2B). Although spectroscopic measurements can overestimate the length of lag phase, significant differences in both maximum growth rate and length of lag periods between *rpoC^WT^* and *rpoC^A494V^* strains conclusively prove the slow-growth phenotype of *rpoC^A494V^* mutants.

**FIG 3:**
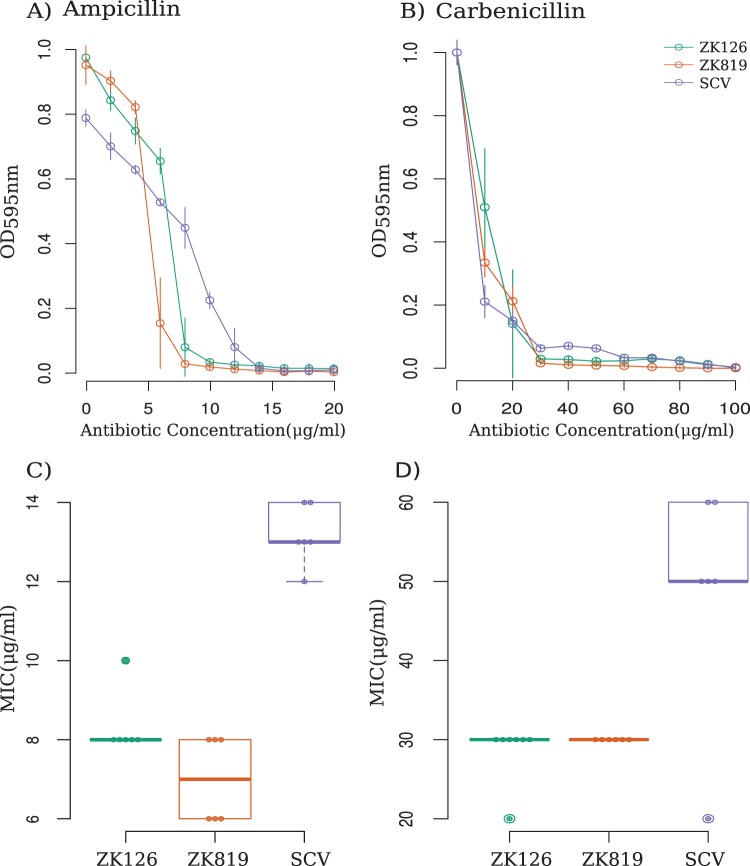
SC exhibits tolerance against beta-lactam antibiotics. (A and B) Kill dynamics of ampicillin (A) and carbenicillin (B) against SC and ancestor strains. (C and D) Distribution of MICs of evolved mutant (SCV), evolutionary ancestor (ZK819), and parent of evolutionary ancestor (ZK126) strains against ampicillin (C) and carbenicillin (D) (*n* = 6).

Small colony variant populations isolated in other bacteria—often in pathogenic contexts—show increased tolerance to antibiotics (15, 16). We tested if this would apply to the SC isolated here by comparing the MICs of three different antibiotics—ampicillin, kanamycin, and ciprofloxacin—against SC, ZK126, and ZK819. SC showed an increased resistance to ampicillin and the analogous carboxypenicillin antibiotic carbenicillin but not to the other two antibiotics (Fig. 3 and Fig. S1).

10.1128/mSphere.00092-20.1 FIG S1 MICs of SCV, ZK819, and ZK126 against kanamycin and ciprofloxacin. Distribution of MICs of kanamycin (A) and ciprofloxacin (B) against SCV, ZK819, and ZK126. Kill dynamics of kanamycin (C) and ciprofloxacin (D) against SCV, ZK126, and ZK819. Download FIG S1, PDF file, 0.04 MB. Copyright © 2020 Nandy et al. 2020 Nandy et al. This content is distributed under the terms of the Creative Commons Attribution 4.0 International license.

We observed that ampicillin clears more SC than wild type when present in lower concentrations. At higher concentrations of the antibiotic, this trend is reversed. This type of concentration-dependent variable response requires different subpopulations to have variable affinity to the antibiotic and is known as “heteroresistance” (17).

### SC shows GASP phenotype in a limited time window in prolonged stationary phase.

Growth advantage in stationary phase (GASP) is defined as the ability of a strain to outcompete its ancestor in prolonged stationary phase (4). To examine the ability of SC to show GASP, we competed SC against its ancestor ZK819 using mixed culture competition experiments (4) in fresh LB and in media spent for different periods with the ancestral strain ZK819. In these experiments, the competing strains were inoculated in three different ratios: 1:1,000, 1:1, and 1,000:1.

**FIG 4:**
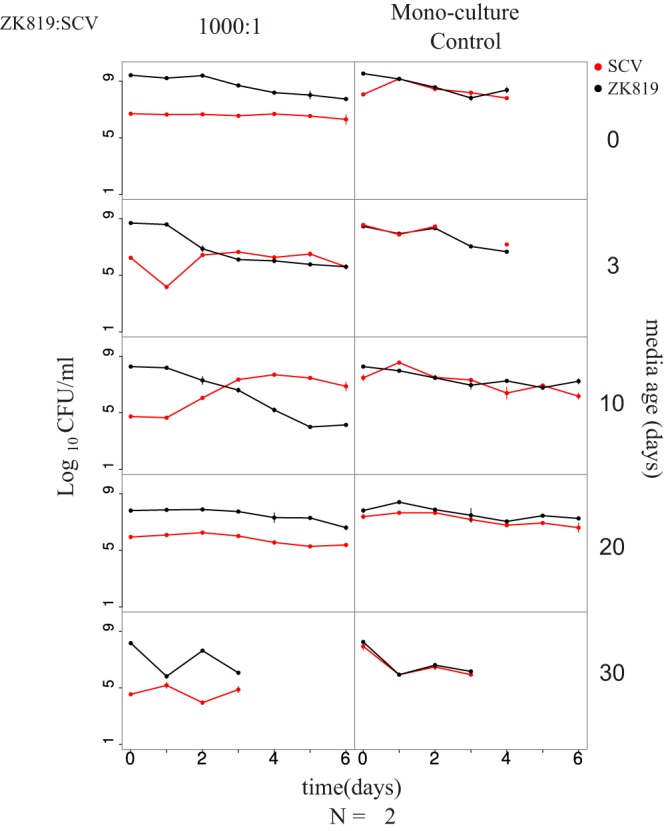
SC exhibits competitive advantage against its ancestor within a specific time window in prolonged stationary phase. Competition dynamics between ZK819 (ancestor) and SC in differently aged LB. Left panel shows competition experiments where ZK819 is inoculated in 1,000-times-higher frequency than SC. Right panel shows competing strains grown individually in the same-age medium as controls. Age of medium used for competition is shown on right. Competition dynamics with other starting frequencies are shown in Fig. S2.

We found that SC does not show any competitive advantage against ZK819 in freshly autoclaved and 3-day-old spent medium. However, it outcompetes ZK819 in 10-day-old medium. Starting from a 1,000-fold minority, SC overtakes ZK819 after 3 days only in the 10-day-old spent medium (Fig. 4). Though it is not unreasonable to expect considerable batch-to-batch variation in spent media across trials, the above result was consistently found across three replicates. In 20-day- and 30-day-old media, however, the competitive advantage of SC was lost, indicating that SC can exhibit GASP only in a narrow time window in prolonged stationary phase (Fig. S2). This is consistent with the idea that the continuous wax and wane of different genetic variants is a reflection of the ever-changing biochemical properties of the prolonged stationary-phase medium (3).

10.1128/mSphere.00092-20.2 FIG S2 Competition dynamics between ZK819 and SCV in different starting frequencies. (Left) Competition flavor when SCV and ZK819 are inoculated in equal proportions (1:1). (Right) SCV inoculated in 1,000-fold-higher proportion than ZK819. Competition performed in differently aged media as shown on right. From top, 0-, 3-, 10-, 20-, and 30-day-old media. Download FIG S2, PDF file, 0.02 MB. Copyright © 2020 Nandy et al. 2020 Nandy et al. This content is distributed under the terms of the Creative Commons Attribution 4.0 International license.

**FIG 5:**
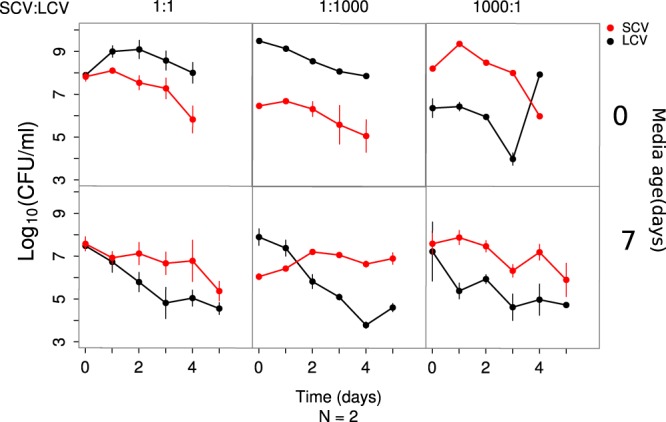
Competitive dynamics between SCV and coexisting strain LCV in fresh (day 0) and 7-day-old spent (day 7) media. Population dynamics in all three possible inoculation ratios (indicated on top) of the competing species are shown in horizontal panels. Competitions performed in 0-day- and 7-day-old media are shown in different rows.

In light of the increasing genetic diversity of the population and the late emergence of SC, it is more reasonable to expect SC to compete with the coexisting LC than with the ancestral ZK819. We therefore investigated the competitive advantage of SC against LC in 10-day-old LB and found that SC, starting from a 1,000-fold minority, overtook LC after 2 days (Fig. 5). Therefore, SC shows the ability to outcompete both ZK819 and LC in prolonged stationary phase under conditions representing a narrow time window.

### *rpoC^A494V^* is a unique mutation in SC that causes small colony size, irrespective of background.

To identify and characterize the genetic basis of the small colony and the GASP phenotype of SC, we performed whole-genome sequencing of SC, LC, and ZK819. We found several mutations common to SC and LC and identified two loci at which the two diverged (for the complete mutation profile of SCV and LCV, please see Table S3). The first was at *rpoS*, encoding σ^S^. Whereas ZK819 carried the attenuated *rpoS819* allele, SC carried what we refer to as *rpoS92. rpoS819* is defined by a 46-bp duplication at the 3′ end of the gene. This duplication results in a longer protein with reduced activity, which may in part be due to its reduced expression at the protein level. *rpoS92* carries a reduplication of the 46-bp region. This introduces a stop codon prematurely for *rpoS819*, resulting in a σ^S^ protein variant of length intermediate between the wild type and *rpoS819*. *rpoS92* partially restores the activity of σ^S^ that had been lost in *rpoS819* (9). LC, in contrast to both SC and ZK819, carries the open reading frame (ORF) of wild-type *rpoS*.

10.1128/mSphere.00092-20.6 TABLE S3 Complete list of mutations identified by whole-genome sequencing of replicate isolates of SCV, LCV, and batch culture population. Whole-genome sequencing was done by sampling respective cultures in stationary phase. Reference genome used to identify mutation Escherichia coli K-12 strain W3110 (GenBank accession no. NC007779.1). Download Table S3, PDF file, 0.2 MB. Copyright © 2020 Nandy et al. 2020 Nandy et al. This content is distributed under the terms of the Creative Commons Attribution 4.0 International license.

The second locus at which these three strains diverged was *rpoC*, coding for the β′ subunit of the core RNA polymerase. Whereas ZK819 and LC carry the wild-type *rpoC* locus, SC has a G:A transition, resulting in the replacement of the alanine at residue 494 by a valine. Mutations in the core RNA polymerase have been found to confer adaptive advantages on E. coli under different conditions and have been found in at least two whole-genome sequencing studies of variants that emerge in prolonged stationary phase (5, 11). We also identified a unique synonymous mutation (H366H) in LC in the gene *icd* coding for isocitrate dehydrogenase, which we have not characterized in this study.

To test the phenotypic effect of *rpoC^A494V^* mutation, we introduced it in different relevant genetic backgrounds using phage-mediated transduction from a *thiC39*::Tn*10* marker (CAG18500), as described in a previous study (18). The genetic backgrounds in which *rpoC^A494V^* was introduced were selected based on their σ^S^ status and their relevance to the strains used or isolated in this study; these were LC (evolved strain, *rpoS^WT^*), ZK819 (ancestor, *rpoS819*), ZK126 (*rpoS^WT^*, the parent of ZK819), and *ΔrpoS* (ZK819::*ΔrpoS*). In addition, the wild-type *rpoC* allele was transduced into SC, reverting the *rpoC^A494V^* mutation to the wild type. The presence of the *rpoC^A494V^* mutation was confirmed by Sanger sequencing with relevant primers (Table S1). Single-end whole-genome sequencing was performed on all transductant strains to check for the presence of any additional indels, copy number variations, and point mutations that may have been inadvertently introduced during transduction and found none apart from those introduced.

**FIG 6:**
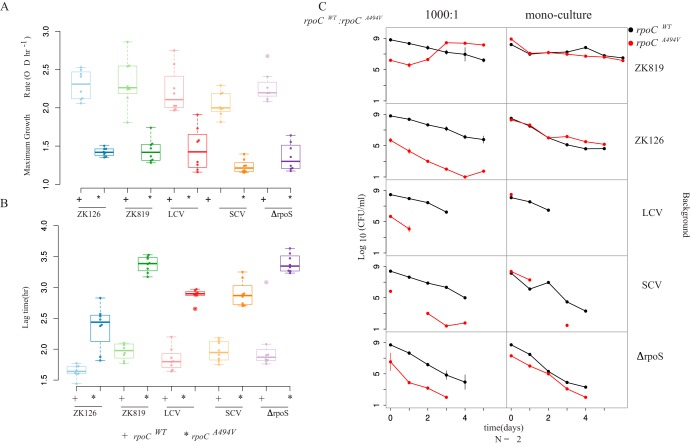
*rpoC^A494V^* is necessary and sufficient to cause a slowdown in growth and confers GASP in a background with attenuated σ^S^ activity. (A) Comparison of maximum growth rates of strains carrying *rpoC^WT^* (marked +) and *rpoC^A494V^* (marked *) in different backgrounds. Maximum growth rates were calculated from growth data as described in Materials and Methods. (B) Comparison of lag times of strains carrying *rpoC^WT^* (+) and *rpoC^A494V^* (*) in different backgrounds. Lag times were calculated from growth curve data as in Materials and Methods. (C) Competition dynamics between strains carrying *rpoC^WT^* and *rpoC^A494V^* in different backgrounds. Left panel shows scenarios where the *rpoC^WT^* strain is inoculated in 1,000-times-higher frequency than the *rpoC^A494V^* strain. Right panel shows competing strains grown individually in the same-age medium as controls. Data shown only for competitions performed in 10-day-old spent medium. Other medium conditions and starting frequencies are provided at https://doi.org/10.6084/m9.figshare.12001212.v1.

We observed that the *rpoC^A494V^*-bearing transductant in all backgrounds, including *ΔrpoS*, produced small colonies on LB agar plates and exhibited slow growth, characteristic of SC in liquid culture (Fig. 6A and B). Conversely, replacing the mutant *rpoC^A494V^* allele in the SC background with the wild-type *rpoC* allele ameliorated slow growth, thus reverting the small colony phenotype to ancestral colony size. These results indicate that the *rpoC^A494V^* mutation is sufficient to cause the small colony phenotype in its bearer, independent of the status of σ^S^.

### The *rpoC^A494V^* mutant might cause inefficient transcription.

The ability of *rpoC^A494V^* to slow growth is independent of the status of σ^S^. This being a mutation in the core RNA polymerase, we suspected that it would have a broad impact on global gene expression levels.

To test whether the mutation causes any alteration to transcription, we tested induction kinetics of the chromosomal *ara* operon in *rpoC* mutant and wild-type strains in the ZK819 background. Note that the *ara* operon comes under the control of the housekeeping sigma factor σ^D^ and not σ^S^. We measured the induction of the *araB* gene over time following the addition of arabinose to an exponentially growing culture in fresh LB by performing RT-PCR using primers amplifying a 134-bp region at the 5′ end of the gene (Table S1). To limit any population-level heterogeneity in *ara* induction (19), we used a nearly 100-fold excess of arabinose for induction.

**FIG 7:**
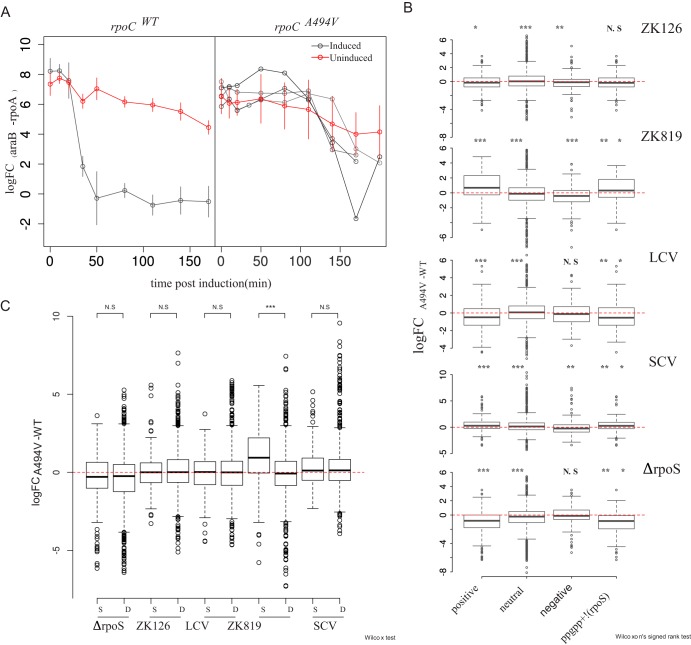
*rpoC^A494V^* mutation affects global gene expression states. (A) Semiquantitative estimation of *araB* expression by RT-PCR in *rpoC^WT^* (left) and *rpoC^A494V^* (right) in the ZK819 background, after induction with 100 mM l-arabinose. Error bars indicate standard deviations (*n* = 4). Individual replicates have been shown separately for the *rpoC^A494V^* strain to emphasize the biological variation observed in the induction pattern in mutant strain compared to wild type. Threshold cycle values from *araB* mRNA were normalized with that of constitutively expressing *rpoA* gene. *araB* measurements from uninduced cultures as controls are shown in red. (B) Differential expression of positive, negative, and neutrally regulated targets of ppGpp between *rpoC^A494V^* and *rpoC^WT^* in different backgrounds (noted in top right of each figure). To control for the behavior of a large number of genes controlled by both σ^S^ and ppGpp, differential expression of genes which are upregulated by ppGpp but are not σ^S^ targets was taken into account. (C) Differential expression of σ^S^ and σ^D^ target genes between *rpoC^A494V^* and *rpoC^WT^* in different backgrounds. Wilcoxon’s test was used to test significance of the difference in median expression values. *P* value scale: ***, <0.001; **, <0.01; *, <0.05.

In the *rpoC^WT^* mutant, *araB* levels increase steeply (difference in *C_T_* values between mutant and wild-type strains in induced and uninduced conditions [ΔΔFC_induced-uninduced_] = −6.05) within 50 min of adding arabinose. In the *rpoC^A494V^* mutant, the increase in mRNA is muted (ΔΔFCinduced-uninduced = −0.74 to −4.54), more variable across biological replicates, and delayed, occurring after 110 min postinduction (Fig. 7A). This delayed and repressed response to arabinose induction in the *rpoC^A494V^* mutant suggests that the mutation reduces the activity of RNA polymerase (RNAP). This is unlikely to be an effect of a possible reduced arabinose transporter expression in the mutant: transcriptome sequencing (RNA-seq) data (see below) showed no significant difference in the basal expression level of *araE* between the wild-type and mutant *rpoC* strains. Thus, slow transcription might underpin the ability of *rpoC^A494V^* to cause slow growth.

### The competitive advantage conferred by the *rpoC^A494V^* mutation depends on the status of σ^S^.

Having established that the *rpoC^A494V^* mutation can cause the small colony phenotype independently of the status of σ^S^, we investigated the ability of this mutation to confer GASP on its bearer. To do this, we performed mixed culture competition experiments of *rpoC^A494V^* strains against *rpoC^WT^* strains in all relevant genetic backgrounds in both fresh and spent media.

We found that *rpoC^A494V^* confers GASP on its bearer only in the ZK819 background, carrying the attenuated *rpoS819* allele, and not others (Fig. 6C) (https://doi.org/10.6084/m9.figshare.12001212.v1). In 10-day-old spent medium, ZK819+*rpoC^A494V^* overtook ZK819+*rpoC^WT^* from a 1,000-fold-lower starting frequency after 2 days. However, SC with *rpoS92* and *rpoC^A494V^* did not outcompete SC with *rpoS92* and *rpoC^WT^*. This suggests, under the assumption that these mutations emerged under a regime of natural selection, that the *rpoC^A494V^* mutant might have arisen in the *rpoS819* background, with *rpoS92* emerging later. We also found that in the ZK819*ΔrpoS* background, both *rpoC^A494V^* and *rpoC^WT^* exhibited precipitous drops in cell count with no viable colonies remaining after 3 days of growth, highlighting the importance of *rpoS* in survival and growth under nutrient-depleted/altered conditions (https://doi.org/10.6084/m9.figshare.12001212.v1 [*ΔrpoS* background]).

These data suggest that *rpoC^A494V^* confers GASP only when σ^S^ is severely attenuated, as seen in *rpoS819*. However, its ability to confer GASP does require some level of functional σ^S^, bringing about the suggestion that it acts through σ^S^.

### *rpoC^A494V^* induces expression of the σ^S^ regulon depending on the *rpoS* genetic background.

Toward understanding the mechanisms by which *rpoC^A494V^* causes the small colony and the GASP phenotypes, we performed RNA-seq-based transcriptome experiments comparing *rpoC^A494V^* and *rpoC^WT^* in different genetic backgrounds differing in their σ^S^ activities (ZK126, ZK819, SC, LC, and *ΔrpoS*). We identified differentially expressed genes that showed a log_2_ fold change of over 1.5 at a *P* value of less than 0.05.

The number of differentially expressed genes across these five backgrounds ranges from 407 to 654. However, the number of genes differentially expressed irrespective of the genetic background was only 18. This shows that the effect of *rpoC^A494V^* on the transcriptome is dependent on the background expression state established at least in part by σ^S^ activity.

Because the ability of *rpoC^A494V^* to confer GASP—as well as alter gene expression states—seemed to depend on the status of σ^S^, we tested the effect of the mutation on the expression levels of σ^S^ targets. Toward this, we compared the fold changes—between *rpoC^A494V^* and *rpoC^WT^*—of targets of σ^S^ with those of a set of targets of the housekeeping sigma factor σ^D^. In a second analysis, we tested for the overlap between σ^S^ and σ^D^ target genes that are differentially expressed in *rpoC^A494V^* over *rpoC^WT^*. When we looked at all genes, σ^S^ targets showed higher positive fold changes in *rpoC^A494V^* than *rpoC^WT^* only in the ZK819 background carrying the attenuated and elongated *rpoS819* allele (Fig. 7B). Consistent with this, sets of differentially expressed gene sets between *rpoC^A494V^* and *rpoC^WT^* were significantly enriched for σ^S^ targets in the ZK819 (*rpoS819*) background, but not the other backgrounds (data available at https://doi.org/10.6084/m9.figshare.12005166.v1).

A previous study had identified *rpoC^A494V^* as a mutation that enables RNAP to bypass the requirement of the small molecule ppGpp in transcribing a ppGpp-dependent, σ^54^ promoter (18). Following from this work, we analyzed the expression patterns of genes known to respond to ppGpp (20) in *rpoC^A494V^* compared to *rpoC^WT^*. We observed that positively regulated targets of ppGpp are upregulated and negatively regulated targets are downregulated in *rpoC^A494V^* compared to *rpoC^WT^* in the ZK819 and SC backgrounds, which harbor elongated *rpoS* alleles with less than wild-type σ^S^ activity (Fig. 7C). This applies for genes that are known to respond to ppGpp but not known to be regulated by σ^S^ as well. While this might indicate that this effect may not be mediated by σ^S^, we observed an opposite pattern in *rpoC^A494V^* compared to *rpoC^WT^* in the *ΔrpoS* background (Fig. 7C). We did not observe any significant effect on ppGpp targets in the *rpoS^WT^* background.

**FIG 8:**
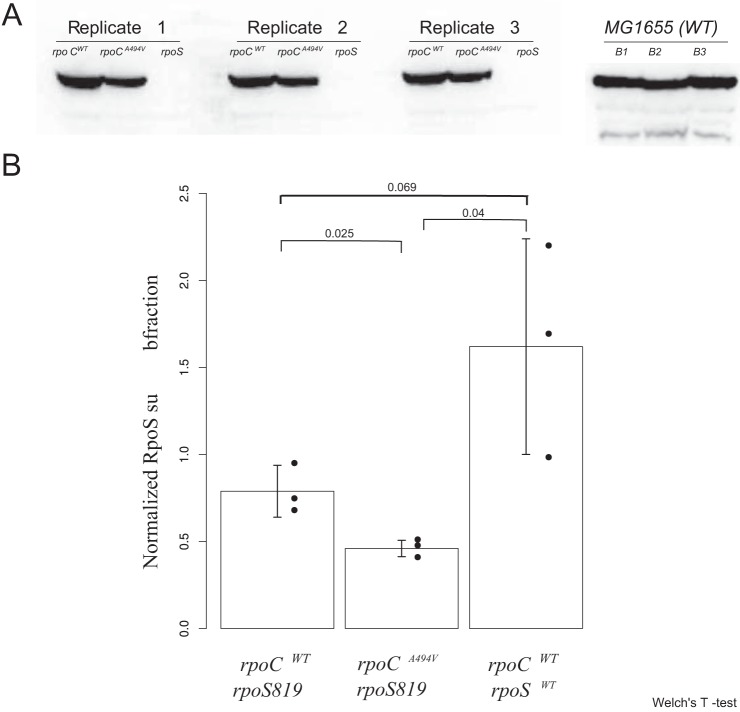
σ^S^ protein level in *rpoC^WT^* and *rpoC^A494V^* strains in ZK819 background. (A) Nitrocellulose blot containing σ^S^ protein in *rpoC^WT^* and *rpoC^A494V^* strains in the *rpoS819* background. The *ΔrpoS* strain, with the deletion introduced into ZK819, is shown as a control. On the right, σ^S^ protein derived from the wild-type MG1655 strain containing the wild-type *rpoS* allele is shown as positive control. A part of the gel was stained with Coomassie blue, and total protein intensities from each lane were quantified, which were then used for normalizing the RpoS protein intensity (Fig. S3). (B) Normalized intensity of σ^S^ protein was quantified for each strain, and average values are shown. Error bars represent standard deviations over three replicates. Statistical significance of difference of intensity between each pair of strains was calculated by Welch’s *t* test, and *P* values are shown.

*rpoC^A494V^* does not seem to induce the σ^S^ regulon by increasing the expression levels of σ^S^, on the basis of our observation that there is little differential expression of the *rpoS* gene in the RNA-seq data, nor is there any increase in σ^S^ protein levels as measured by Western blotting. In fact, Western blots indicate a decrease in the levels of σ^S^ in *rpoC^A494V^* (Fig. 8). This suggests that *rpoC^A494V^* may activate the σ^S^ regulon by affecting σ-factor competition for the core RNA polymerase. There is evidence (21) that ppGpp reduces the ability of σ^D^ to compete with at least two alternative sigma factors, including σ^S^. Whether the induction of the σ^S^ regulon by *rpoC^A494V^* operates through this mechanism, and whether this might even require the extended C-terminal tail that *rpoS819* and *rpoS92* alleles add to the wild-type *rpoS*, remains to be tested.

10.1128/mSphere.00092-20.3 FIG S3 (A) Overlay of RpoS protein on nitrocellulose membrane indicating ladder. *rpoC^A494V^*- and *rpoC^WT^*-harboring strains are on the left; E. coli MG1655 is shown on the right. (B) Coomassie blue-stained protein gel showing protein bands (>50 kDa) from whole-cell extracts of different strains. Net intensity for each lane was used to normalize RpoS band intensity for that lane. Download FIG S3, PDF file, 0.5 MB. Copyright © 2020 Nandy et al. 2020 Nandy et al. This content is distributed under the terms of the Creative Commons Attribution 4.0 International license.

In summary, though *rpoC^A494V^* increases the expression of the σ^S^ regulon in backgrounds with low σ^S^ activity, this effect requires the presence and expression of σ^S^. The manifestation of GASP in the regime in which *rpoC^A494V^* displays a selective advantage might require higher σ^S^ activity, in contrast to earlier time points in which attenuated variants of σ^S^ such as *rpoS819* are favored. In backgrounds with elongated *rpoS* alleles (those with extended C-terminal ends, i.e., *rpoS819* and *rpoS92*), the effect of the *rpoC^A494V^* allele on the transcriptome shows similarities to that of higher intracellular ppGpp. This effect also seems to be mediated by σ^S^ as in the *ΔrpoS* background; the effect of the *rpoC^A494V^* mutation flips and resembles that of lower intracellular ppGpp.

## DISCUSSION

This work highlights the global effect of a single nucleotide substitution in the β′ subunit of bacterial RNA polymerase emerging a few weeks into prolonged stationary phase. We show that in this phase, slow growth is advantageous within a certain time window. Our experiments demonstrate that the *rpoC^A494V^* mutation is necessary and sufficient to confer slow growth on the bacteria. However, to show the GASP phenotype, an attenuated *rpoS819* allele is additionally required.

Genetic and phenotypic diversification of an initially isogenic inoculum is a characteristic of prolonged stationary phase. Although we cannot reject the assumption that some alleles gaining prominence up to detection levels later in long-term stationary phase (LTSP) could be present in the initial inoculum, we know novel mutations arise in prolonged stationary phase based on the ambient environmental conditions and largely under selection (5).

Constantly changing physicochemical conditions of the media result in different subpopulations arising over time. This is orchestrated by different allelic forms of σ^S^ that sequentially emerge with time. Within the first 10 days, the elongated and attenuated *rpoS819* allele emerges, giving rise to the first GASP population (4). A constant competition for occupying the RNA polymerase holoenzyme exists between the housekeeping sigma factor σ^D^ driving growth and the stationary-phase sigma factor σ^S^ involved in dormancy and maintenance (22, 23). There is always a trade-off between growth and stress response, which is determined by this competition between the two sigma factors. The expression and activity of σ^S^ are tightly regulated at multiple levels, and adjusting this switch helps the bacterial cell modulate global gene expression states according to environmental cues. (24, 25). Additionally, the gene for σ^S^ is highly mutable, thus enabling genetic alterations of the growth-stress tolerance trade-off (26).

The attenuated *rpoS819* allele alters this growth-stress response trade-off in favor of growth. In our experiments, we found that the *rpoS819* allele, when evolved in LB medium, suffers another reduplication at the C-terminal end around 19 to 22 days postinoculation, giving rise to the *rpoS92* allele, which has activity closer to that of the wild-type *rpoS* allele (9). The *rpoC^A494V^* mutation also enhances the activity of σ^S^. This suggests that stress response takes some precedence in later stages of stationary phase. GASP outcomes depend on initial medium conditions (27), and this can interplay with the balance between stress tolerance and growth.

**FIG 9:**
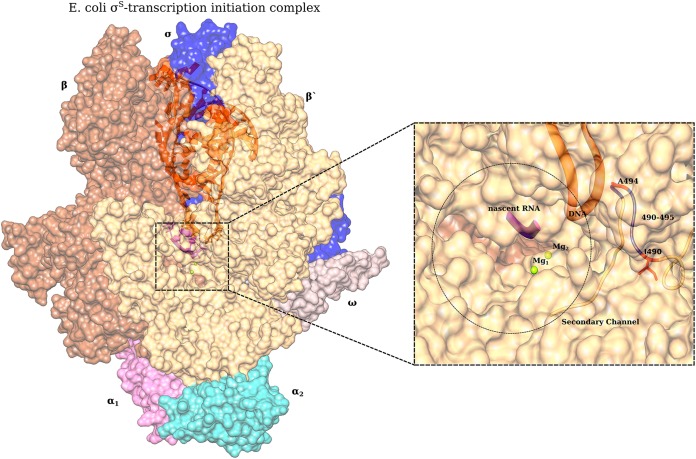
Structural representation of E. coli RNA polymerase holoenzyme (PDB 5IPL) along with DNA (orange) and nascent RNA (purple). (Inset) Secondary channel and the locus of interest A494 along with other reported mutations known to cause slow transcription are indicated: A490E (25) and Δ490–495 (26). This figure was generated by Aravind Ravichandran.

Mutations in the *rpoABC* operon result in a wide variety of pleiotropic phenotypes, by affecting different steps of the transcription initiation process (28). In a study by Mukhopadhyay et al., mutations mapping near the locus of our mutation of interest on the β′ subunit were found to confer resistance to antibacterial micropeptide microcin J25 (29). This region lies in the secondary channel of RNA polymerase in the shape of a hollow cylinder. Our mutation of interest *rpoC^A494V^* could alter the local architecture of the secondary channel, reducing the rate of influx of ribonucleotides to be added to a nascent RNA chain at the catalytic site of RNA polymerase. Other mutations like A490E (30) and Δ490–495 (31) in RpoC alter nucleoside triphosphate (NTP) sensing and uptake and finally to delay stable elongation complex (SEC) formation (Fig. 9). This would lead to slow transcription by the mutated polymerase and explain the slow growth exhibited by *rpoC^A494V^* in different *rpoS* backgrounds. Further, this slow transcription could internally trigger a stress response, initiating the upregulation of σ^S^ targets that we observe in ZK819 and SC backgrounds. Bacterial sigma factors are known to interact with the β and β′ subunits of RNA polymerase (32), and recently, it has been shown that σ^S^ directly interacts with the β′ subunit of RNA polymerase, diminishing the pore size of the secondary channel (32). This interaction could be affected by different allelic variants of *rpoS* (*rpoS819* and *rpoS92*).

In recent literature, there has been a substantial focus on SC of pathogenic origin. Different strains of Staphylococcus aureus (33), Pseudomonas aeruginosa (34), Burkholderia cepacia (35), and E. coli (36), have been described to form small colony variants, some of which are genetically stable. Among the genetically stable variants of SC, most are metabolic mutants, defective either in the electron transport chain or in the thymidine biosynthesis pathway (14). Early studies have found nonpathogenic E. coli to unstably form small colonies under prolonged exposure to chemicals like phenol or anthraquinone (37, 38). Under our experimental framework, we observed that E. coli diversifies into stable, regulatory mutants which phenotypically produce small colonies with enhanced stress tolerance after 3 weeks of incubation in rich spent medium.

In summary, we show that a mutation in the core RNA polymerase provides a competitive advantage to its bearer in deep stationary phase, by altering the balance between growth and stress tolerance.

## MATERIALS AND METHODS

### Bacterial strains and culture conditions.

Escherichia coli K-12 strain W3110 is the parent strain of Escherichia coli ZK126. The latter, when evolved in prolonged stationary phase for 10 days, gave rise to Escherichia coli ZK819, which has an elongated and attenuated *rpoS* allele (4) called *rpoS819*. ZK819 was evolved in prolonged stationary phase for 28 days (5). The small colony form isolated is referred to as SC, and its coexisting ancestral-colony-sized variant is referred to as LC. Strains are described in Table 1.

**TABLE 1:** Strains used in this study

Strain	Description	Source or reference
Escherichia coli W3110	Wild-type strain	CGSC 4474
ZK126	W3110 Δ*lac U169 tna2*, parent of ZK819	5
ZK819	ZK126 *rpoS819*; evolutionary ancestor of SCV and LCV	5
Large colony variant (LCV)	Evolved strain, colony size comparable to wild-type strain, *rpoS^WT^ icd^H366H^*	This study
Small colony variant (SCV)	Evolved strain, small colony size, *rpoS92 rpoC^A494V^*	This study

Additional long-term stationary-phase experiments were performed to test the repeatability of the appearance of small colony forms. Twelve replicate lines of E. coli were propagated in 500 ml of LB in 1.5-liter Erlenmeyer flasks. They were grown at 37°C with circular agitation at 200 rpm. Every day, 1-ml aliquots were taken from each flask. Five hundred microliters of this sample was used to prepare dilutions in an 0.9% NaCl solution which were then plated on LB plates in two technical replicates. The remaining 500 μl solution was mixed with an equal volume of 50% glycerol and saved at −80°C as stocks (Fig. 1). The plates were incubated at 37°C for 16 to 18 h, and the colonies were counted to track population changes over time and to check for contamination. Images were taken with the help of the Gel-Doc system. Putative small colonies, whenever seen, were restreaked to verify the stability of the phenotype, and a separate count of them was maintained daily.

For measuring growth characteristics of various strains, 5 ml of LB in a 50-ml centrifuge tube (Tarsons) was inoculated with single colonies of respective strains, grown for 12 to 16 h at 37°C, and agitated at 200 rpm to form the starter culture. This culture was diluted 1,000-fold in 30 ml LB contained in 100-ml Erlenmeyer flasks (Pyrex; NC-0400). Cultures were maintained at 37°C, with circular shaking at 200 rpm to maintain aeration. Growth dynamics were tested in rich medium (Luria broth [HiMedia], containing 5 g/liter NaCl), as well as minimal medium (M9) with 0.5% glucose as carbon source. Bacterial growth was measured by tracking change in optical density (OD) at 600 nm.

### Calculation of maximum growth rate and lag time.

To estimate the maximum growth rate, specific growth rates of each strain were calculated from turbidimetric change for every 15 min over 20 h. Based on the extent of initial noise, a sliding window of 5 to 8 time points in size was taken for each strain, and the median growth rate was ascertained for each window. The element of the window showing maximum median was defined as corresponding to the maximum growth rate for a specific strain.

To calculate time spent by each culture in lag phase, the last point of lag was determined according to the method of Bertrand (39). The intersection of nongrowing lag phase and exponentially growing log phase was ascertained by fitting linear models to growth dynamics. The time obtained from this intersecting point was considered the duration of the lag phase for a specific strain.

### Construction of strains with *rpoC^A494V^* mutation.

Transferring and reverting the point mutation *rpoC^A494V^* in different genetic backgrounds were performed using Escherichia coli strain CAG18500 (CGSC; *thiC39*) (18). In this strain, *thiC*, a gene 4.6 kb upstream of *rpoC*, is tagged with Tn*10* carrying a tetracycline resistance marker. The cotransduction frequency of *rpoC* in this strain has been previously reported as 65% (18). P1 transduction, using this strain as donor, was used to first transfer the selection marker to the *rpoC^A494V^*-carrying strain. Among transductants having the selection marker, one set carried *rpoC^A494V^* and the rest *rpoC^WT^*. The former were used as donors for further P1 transduction of the mutation into different genetic backgrounds. The transductants were plated on LB agar having 12.5 mg/ml of tetracycline and were screened for the presence or reversion of the point mutation by Sanger sequencing (see Table S2 in the supplemental material). Marked strains carrying the wild-type *rpoC* gene were utilized as donor to revert the mutant allele where applicable.

### Determination of MICs of different antibiotics against SC and ancestor strains.

Stock solutions (100 μg/ml) of relevant antibiotics were prepared, and serial dilutions were made for each antibiotic within a range of 0 to 100 μg/ml in 300 μl of LB. Two hundred microliters of LB with the appropriate antibiotics was dispensed into wells of a 96-well plate, which was then inoculated with overnight-grown cultures of appropriate strains in 1:100 dilution. The plates were incubated for 24 h at 37°C, 200 rpm. The turbidity of the cultures was checked by a spectrophotometer at 600 nm. OD at 600 nm was plotted as a function of antibiotic concentration, and the lowest concentration of the antibiotic which produced no perceptible growth at a threshold of OD 0.05 was taken as the MIC for that antibiotic. The experiment was repeated over 6 independent replicates for each strain.

Additionally, LB-agar plates containing a specific antibiotic were prepared at concentrations ranging from 0 to 100 μg/ml. Overnight-grown cultures were diluted 6- to 8 fold in saline solution and plated on LB plates with different antibiotic concentrations. After incubation at 37°C for 16 to 18 h, the resulting colonies were counted. From each plate, the number of CFU was determined per unit volume and the same was plotted as a function of antibiotic concentrations. The lowest concentration of antibiotic which did not exhibit any colony growth was taken as the MIC of that antibiotic for a specific strain.

### Competition experiment in fresh and spent media.

Mixed culture competition experiments (4) were carried out to test the fitness advantage of the mutant strain compared to wild-type strains in different backgrounds. Competing strains were tagged with resistance markers for kanamycin or tetracycline in a featureless genomic region between *htrC* and *rpoC*. They were then inoculated in 5 ml of LB without antibiotics in 50-ml centrifuge tubes. Competing strains were inoculated either in equal frequencies (500 μl of each in 5 ml LB) or with one of them in 1,000-fold excess (1 ml and 1 μl in 5 ml medium). As a control, they were inoculated in isolation to probe population dynamics under no competition. These centrifuge tubes were then maintained for 5 days under standard growth conditions. Every day, cultures were appropriately diluted in saline and plated on kanamycin (50 μg/ml)- and tetracycline (12.5 μg/ml)-supplemented LB agar plates. Colonies developed after overnight incubation at 37°C were counted.

In addition to freshly autoclaved medium, competition experiments were carried out in medium spent by the ancestor ZK819. Three-, 10-, 20-, and 30-day-old media were used for competitions for all backgrounds. To prepare spent media, 500 ml of LB in a 1-liter flask was inoculated with overnight-grown culture of ZK819 in 1:100 dilution. This flask was incubated at 37°C, with circular shaking at 200 rpm. After appropriate growth, 100 ml of the culture was taken from the flask. Bacterial cells were pelleted by spinning the culture at 4,000 rpm for 15 min. The supernatant was then passed through a 0.45-μm membrane filter (Sartorius) and stored in sterile 500-ml bottles.

### Arabinose induction and estimation of *araB* mRNA by qPCR.

Appropriate cultures were incubated overnight in rich medium and were diluted 100-fold in 100 ml of LB. They were allowed to grow until mid-exponential phase. Induction was performed at maximum growth rate stage with l-arabinose (HiMedia) added to a final concentration of 100 mM in the culture. Transcription “Stop” solution was prepared according to the method of Vogel et al. and prechilled in a −30°C bath (40). Postinduction, 500-μl samples were periodically collected and mixed with equal volumes of stop solution in a −30°C bath. After a 30-min incubation, a whole-RNA fraction was isolated from each sample by TRIzol (Ambion)-based extraction.

qPCR was performed to amplify specific regions of *araB* and *rpoA* transcripts with relevant primers (Table S1). The One Step TB green PrimeScript RT-PCR kit II (TaKaRa, RR086B) was used for qPCR on the QuantStudio 6 Flex system (Applied Biosystems). Fifty micrograms of total RNA was used for each reaction. *araB* transcripts were estimated and internally normalized by *rpoA* transcripts.

### Whole-genome and whole-transcriptome sequencing.

Whole genomes of SC, LC, and ZK819 were isolated from single colonies in replicates and grown overnight in LB. Whole-genome isolation was performed using the GenElute bacterial DNA isolation kit (Sigma-Aldrich). Paired-end sequencing was done by using the Illumina MiSeq platform; libraries for the same were prepared with the TruSeq DNA Nano library preparation kit (Illumina) as suggested by the manufacturers.

Sequence data were mapped to the reference sequence of Escherichia coli K-12 strain W3110 (GenBank accession no. NC007779.1). The Breseq pipeline (41; https://barricklab.org/twiki/bin/view/Lab/ToolsBacterialGenomeResequencing), which uses Bowtie for read alignment, was used to call single nucleotide polymorphisms (SNPs), indels, and structural variants (33). Mutations present in only SC at 100% frequency and not present in either LC or ZK819 were considered unique to SC.

To verify whether *rpoC^A494V^* transductant strains harbored only the said mutation compared to donors, whole genomes of transductants from five different *rpoS* backgrounds were sequenced. *rpoC^WT^* and *rpoC^A494V^* strains in ZK126, ZK819, LC, SC, and *ΔrpoS* backgrounds were sequenced in duplicates in two different stages of growth. Cells were isolated during maximum growth rate, which occurs just before the population enters exponential growth, and during the stationary phase of growth.

To look at the effects of the *rpoC^A494V^* mutation on gene expression, whole-RNA fractions were isolated from the cell lysate of wild-type and *rpoC^A494V^* transductants in different backgrounds. Whole-RNA isolation was performed after the bacterial cultures entered stationary phase, upon 16 h of growth. RNA isolation was performed using TRIzol reagent (Ambion). The mRNA fraction from whole RNA was enriched using the MICROBExpress kit (Ambion). The purity of whole RNA was checked by assessing the *A*_260_/*A*_280_ ratio in NanoDrop and further on Bioanalyzer. rRNA contamination was checked on a 1% agarose gel and on Bioanalyzer. Sequencing was carried out on an Illumina HiSeq2500 platform, and library preparation was done using the TruSeq stranded mRNA library preparation kit (Illumina).

RNA-seq data were aligned to the genome of Escherichia coli K-12 strain W3110 (GenBank accession no. NC007779.1) using BWA (42). Read coverage per gene was calculated using SAMtools (v.1.2) and BEDTools (v2.25.0). EdgeR pipeline (Bioconductor) was used to find differentially expressed genes in mutants (*rpoC^A494V^*) compared to wild type (*rpoC^WT^*) in different backgrounds. Normalization and calling of differentially expressed genes were done according to the method of Chen et al. (43). A *P* value cutoff of 0.05 was applied to identify differentially expressed genes.

### Estimating σ^S^ levels with Western blotting.

Intracellular levels of *rpoS* protein were estimated between *rpoC^WT^* and *rpoC^A494V^* strains in the ZK819 background. Whole protein was isolated from respective cultures grown overnight by trichloroacetic acid (TCA) extraction. One hundred micrograms of whole-cell lysate from each strain was run on a NuPage 4 to 12% Bis-Tris protein gel (Thermo Scientific) at 120 V for 3 h. *rpoS* protein was probed by anti-mouse IgG anti-E. coli RNA σ^S^ clone-1RS1 antibody (BioLegend; batch B274469). The protein gel was cut at the 50-kDa ladder mark. One part was used to probe the protein of interest by transferring to a nitrocellulose membrane, and the rest was stained with Coomassie blue and imaged under UV fluorescence. Horseradish peroxidase (HRP)-conjugated IgG (Sigma) was used to develop the blot. HRP signal was detected using SuperSignal West Dura extended-duration substrate (Thermo Scientific). RpoS signal from each lane was normalized by the total protein for that lane.

### Data availability.

RNA sequencing data can be accessed from Gene Expression Omnibus under accession number GSE141883. Whole-genome sequencing data can be accessed from the NCBI Sequence Read Archive under accession number SRP236016.
