# Supplementary material for: A Mutant RNA Polymerase Activates the General Stress Response, Enabling Escherichia coli Adaptation to Late Prolonged Stationary Phase
Source: mSphere. 2020 Apr 15;5(2):e00092-20. doi: 10.1128/mSphere.00092-20 (PMC7160681; doi:10.1128/mSphere.00092-20)
Supplement: TABLE S1 [file mSphere.00092-20-st001.pdf]

Strain	Forward	Reverse
rpoC re-sequencing	5'-TTACTCGTTATCAGAACCGCCAG-3'	5'-TTATTAAAGTTTCTGAAAGCGC-3'
araB mRNA estimation	5'-CAAGCACGGTTTTCAGTG-3'	5'-GATTCTGTGCGAGCTTTGGC-3'
rpoA mRNA estimation	5'-CATCACGTAAGTCAACGAAAGC -3'	5'-GCTGGTCATCGAAATGGAAAC-3'
