# Supplementary material for: A Mutant RNA Polymerase Activates the General Stress Response, Enabling Escherichia coli Adaptation to Late Prolonged Stationary Phase
Source: mSphere. 2020 Apr 15;5(2):e00092-20. doi: 10.1128/mSphere.00092-20 (PMC7160681; doi:10.1128/mSphere.00092-20)
Supplement: TABLE S2 [file mSphere.00092-20-st002.pdf]

Table S2 (Unique mutations in SCV strains isolated from second run of evolution in LTSP)

**Strain**	**Mutation**	**Gene**	**Description**
Strain#2	1. T59P<br>2. L354Q	1. *paaX*<br>2. *lon*	1.DNA-binding transcriptional repressor PaaX<br>2.Lon protease
Strain#8	Insertion of 1,199bp	*gatA*	Rac prophage; uncharacterized protein YdaG
Strain#10	1. Δ13 bp<br>2.H164L	1. *acrB*<br>2.*cpdA*	1.multidrug efflux pump RND permease AcrB<br>2.cAMP phosphodiesterase
Strain#12	Q226*	cpdA	cAMP phosphodiesterase
