# Supplementary figures and images for: A Mutant RNA Polymerase Activates the General Stress Response, Enabling Escherichia coli Adaptation to Late Prolonged Stationary Phase
Source: mSphere. 2020 Apr 15;5(2):e00092-20. doi: 10.1128/mSphere.00092-20 (PMC7160681; doi:10.1128/mSphere.00092-20)

**A Kanamycin**

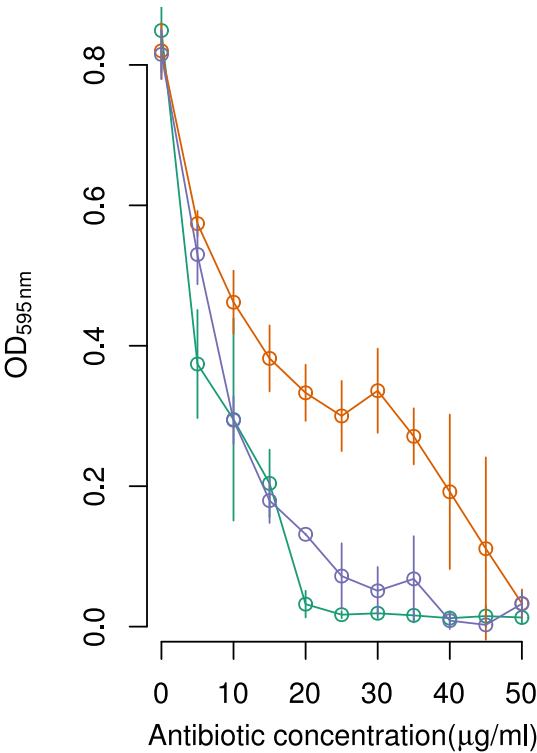

**B Ciprofloxacin**

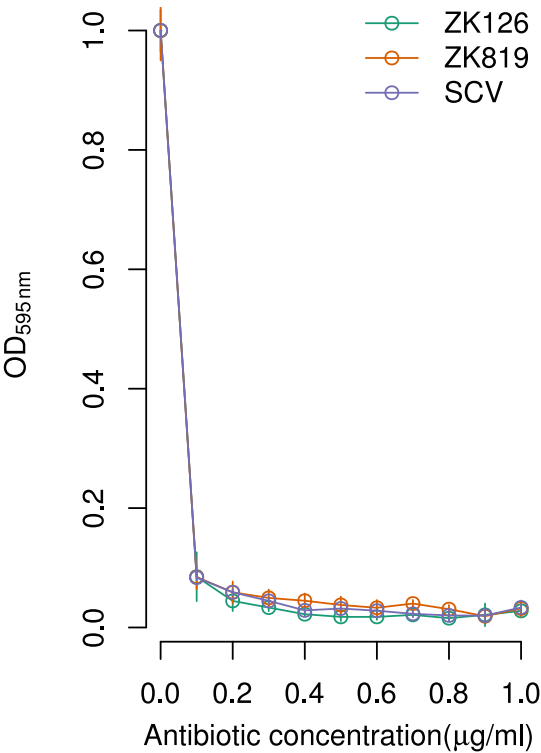

**C**

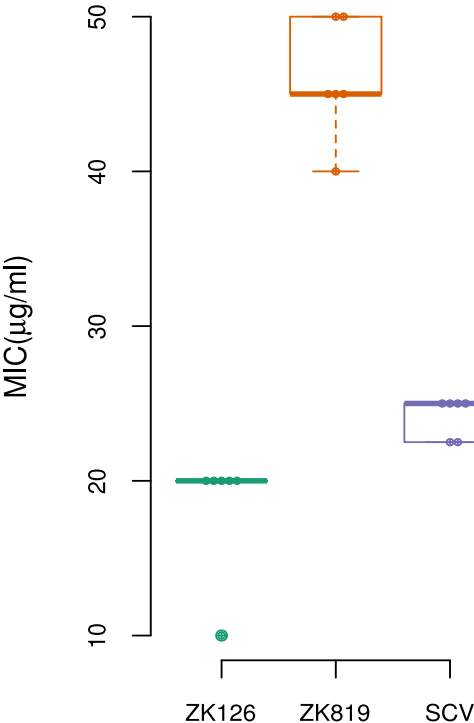

**D**

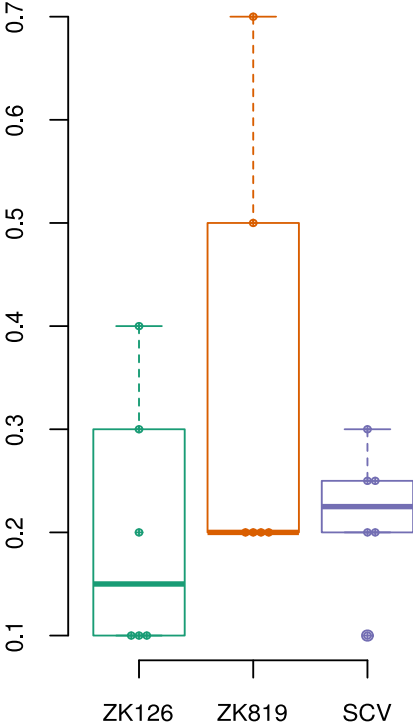

Supplement: FIG S1 [file mSphere.00092-20-sf001.pdf]

ZK819:SCV

1:1

1:1000

● SCV  
● ZK819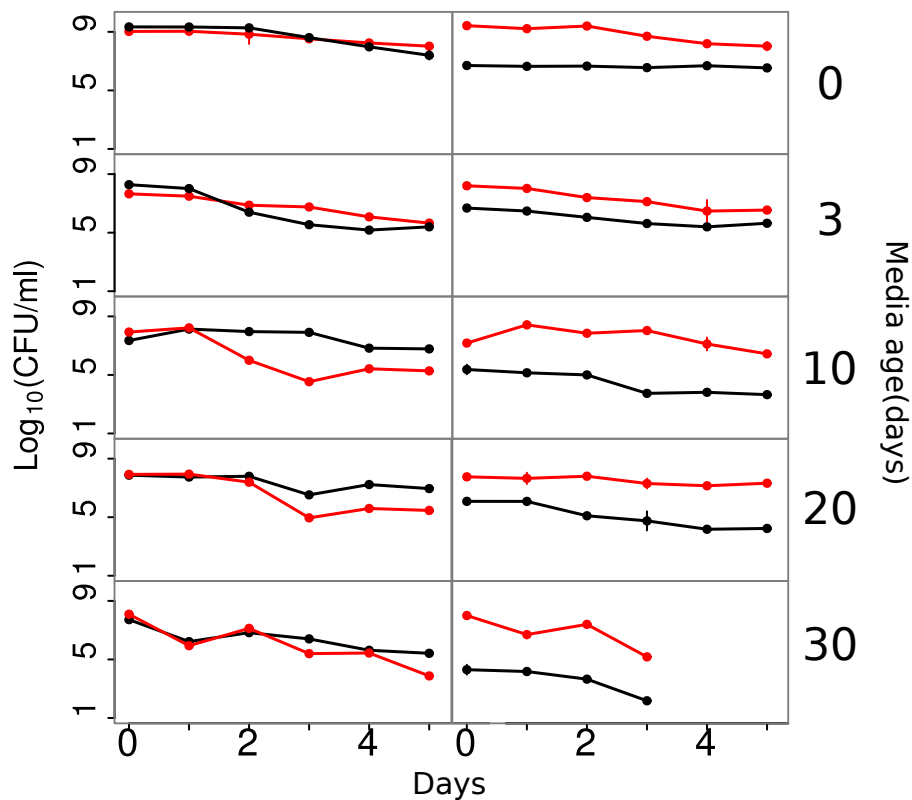

Supplement: FIG S2 [file mSphere.00092-20-sf002.pdf]

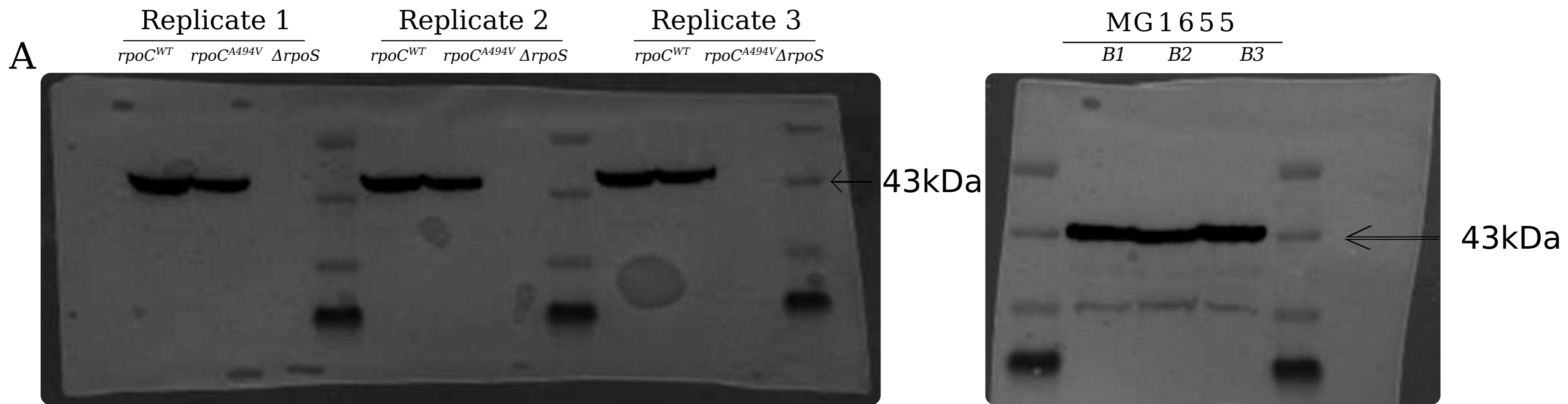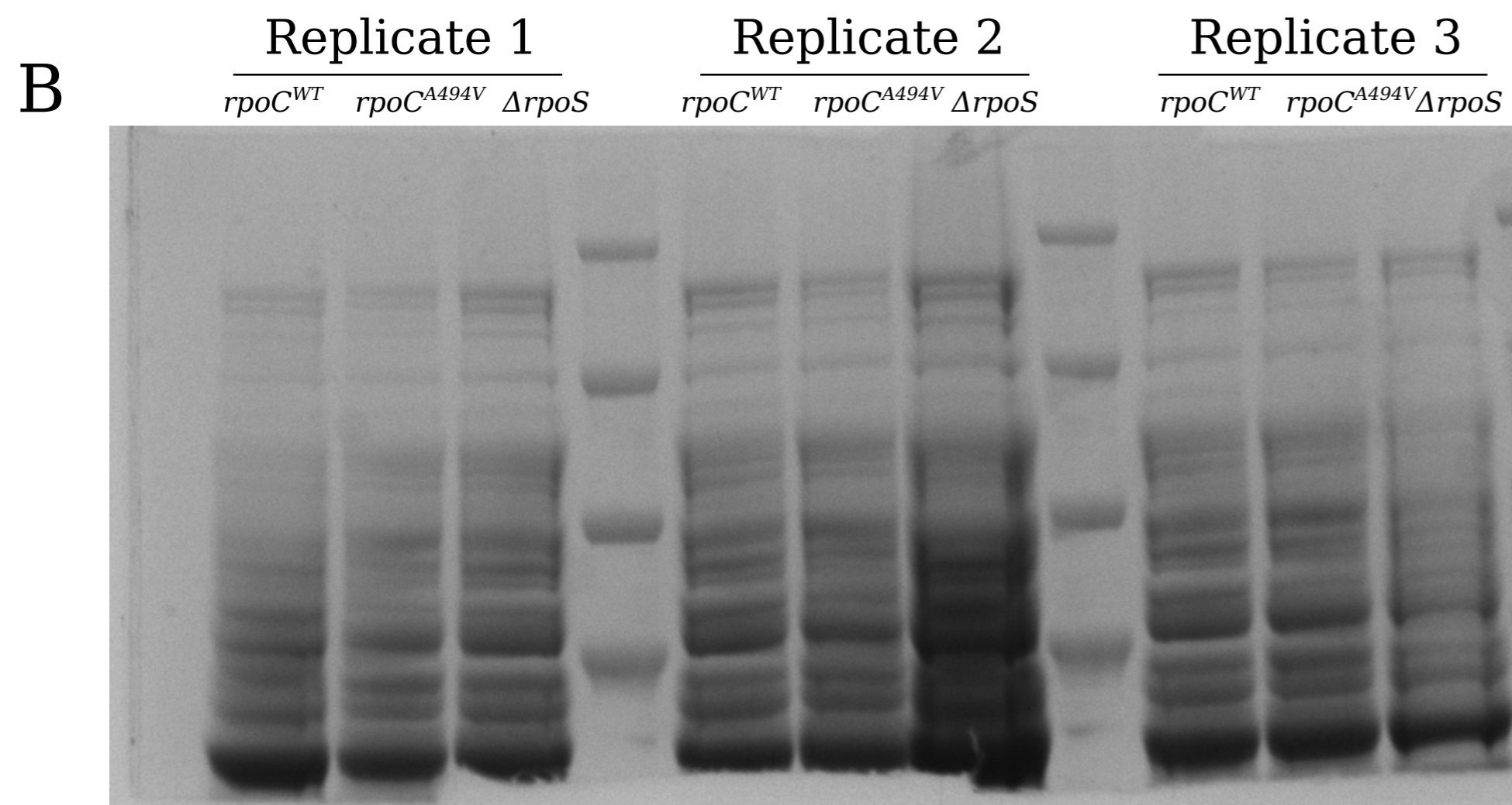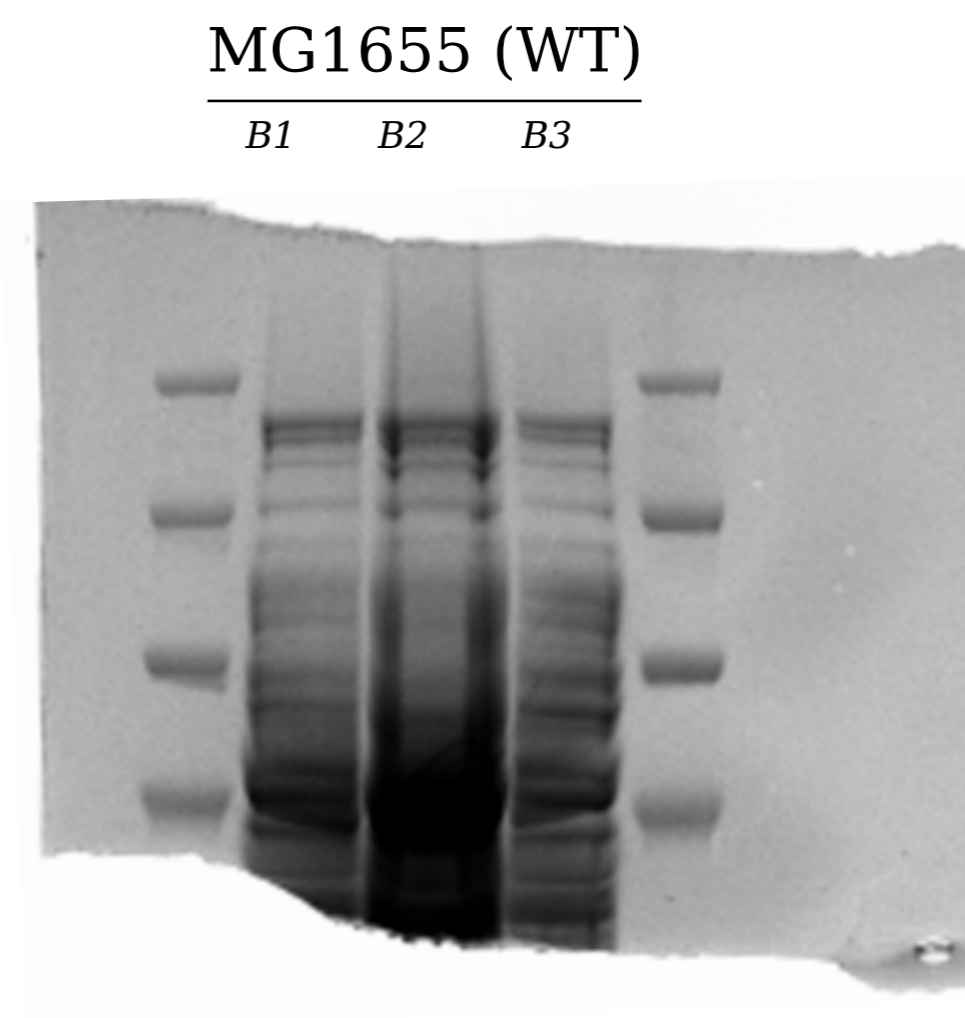

Supplement: FIG S3 [file mSphere.00092-20-sf003.pdf]
