# Supplementary material for: A Mutant RNA Polymerase Activates the General Stress Response, Enabling Escherichia coli Adaptation to Late Prolonged Stationary Phase
Source: mSphere. 2020 Apr 15;5(2):e00092-20. doi: 10.1128/mSphere.00092-20 (PMC7160681; doi:10.1128/mSphere.00092-20)
Supplement: TABLE S3 [file mSphere.00092-20-st003.pdf]

Table T3.1 (SCV, isolate 1)

position	mutation	freq	annotation	gene	description
66457	C→A	100%	V32F (GTT→TTT)	*araD* ←	L-ribulose-5-phosphate 4-epimerase
97880	C→T	100%	S265F (TCC→TTC)	*murD* →	UDP-N-acetylmuramoyl-L-alanine:D-glutamate ligase
167632	C→A	100%	P50Q (CCG→CAG)	*fhuA* →	ferrichrome outer membrane transporter
274547	A→G	100%	intergenic (-206/-2)	*insH* ← / → *mmuP*	IS5 transposase and trans-activator/S-methylmethionine transporter
274731	Δ97,240 bp	100%		*[mmuP]–[mhpD]*	***90 genes [mmuP], mmuM, afuC, afuB, insB, insA, ykgN, yagB, yagA, yagE, yagF, yagG, yagH, yagI, argF, insB, insA, yagJ, yagK, yagL, yagM, yagN, intF, yagP, yagQ, yagR, yagS, yagT, yagU, ykgJ, yagV, yagW, yagX, yagY, yagZ, ykgK, ykgL, Y75_p4288, Y75_p4289, eaeH, insE, insF, ykgA, ykgB, ykgI, ykgC, ykgD, ykgE, ykgF, ykgG, ykgH,***

position	mutation	freq	annotation	gene	description
					betA, betB, betI, betT, yahA, yahB, yahC, yahD, yahE, yahF, yahG, yahH, yahI, yahJ, yahK, yahL, yahM, yahN, yahO, prpR, prpB, prpC, prpD, prpE, codB, codA, cynR, cynT, cynS, cynX, lacA, lacY, lacZ, lacI, mhpR, mhpA, mhpB, mhpC, [mhpD]
547694	A→G	100%	intergenic (+123/-1156)	*fdrA* → / → *ylbF*	acyl-CoA synthetase with NAD(P)-binding Rossmann-fold domain/hypothetical protein
547831	+G	100%	intergenic (+260/-1019)	*fdrA* → / → *ylbF*	acyl-CoA synthetase with NAD(P)-binding Rossmann-fold domain/hypothetical protein
556858	A→T	100%	L36Q (CTG→CAG)	*folD* ←	bifunctional 5,10-methylene--tetrahydrofolate dehydrogenase/ 5,10-methylene--tetrahydrofolate cyclohydrolase
569996	G→C	100%	intergenic (+345/-120)	*ybcK* → / → *ybcL*	recombinase/kinase inhibitor
654214	Δ1,199 bp	100%		*insH*	*insH*

position	mutation	freq	annotation	gene	description
749354	C→A	100%	P17P (CCG→CCT)	*ybgO* ←	fimbrial-like adhesin protein
758440	C→A	100%	F110L (TTC→TTA)	*sdhB* →	succinate dehydrogenase, FeS subunit
887234	C→A	100%	A176A (GCG→GCT)	*ybjJ* ←	transporter
987574	G→T	100%	intergenic (-170/+433)	*ompF* ← / ← *asnS*	outer membrane porin 1a (Ia;b;F)/asparaginyl tRNA synthetase
1093686	T→C	100%	V130A (GTA→GCA)	*ycdT* →	diguanylate cyclase
1097580	C→T	20.7%	intergenic (+329/+226)	*ycdU* → / ← *serX*	inner membrane protein/tRNA-Ser
1170573	C→T	100%	intergenic (-164/-77)	*ycfQ* ← / → *ycfR*	DNA-binding transcriptional regulator/hypothetical protein
1301041	Δ1,336 bp	100%		*insC–insD*	*insC, insD*
1318876	G→A	100%	P21S (CCT→TCT)	*trpA* ←	tryptophan synthase subunit alpha
1413639	Δ23,060 bp	100%		*[ydaO]–ynaE*	***30 genes [ydaO], intR, ydaQ, ydaC, lar, recT, recE, racC, ydaE, kil, sieB, ydaF, ydaG, racR, ydaS, ydaT, ydaU, ydaV, ydaW, rzpR, rzoR, trkG, ynaK, ydaY, ynaA, insH, stfR, tfaR, pinR, ynaE***
1483588	G→A	100%	L322L (CTG→CTA)	*ynbD* →	phosphatase, inner

position	mutation	freq	annotation	gene	description
					membrane protein
1496775	Δ1 bp	100%	coding (11/924 nt)	*ydcI* ←	DNA-binding transcriptional regulator
1615814	C→A	100%	D202Y (GAC→TAC)	*yneI* ←	aldehyde dehydrogenase
1669599	Δ1 bp	100%	coding (680/1221 nt)	*dgsA* ←	DNA-binding transcriptional repressor
1724327	Δ3 bp	12.6%	coding (493-495/2013 nt)	*ydhK* →	inner membrane protein
1844073	G→A	100%	intergenic (-224/-12)	*ynjH* ← / → *gdhA*	hypothetical protein/glutamate dehydrogenase, NADP-specific
1918872	T→A	100%	S301T (TCC→ACC)	*yebS* →	inner membrane protein
1973476	G→A	100%	L310L (CTC→CTT)	*tar* ←	methyl-accepting chemotaxis protein II
2005401	C→T	100%	E115K (GAA→AAA)	*fliC* ←	flagellar filament structural protein
2170094	C→A	100%	intergenic (-437/+32)	*ogrK* ← / ← *yegR*	DNA-binding transcriptional regulator/hypothetical protein
2320795	Δ1,336 bp	100%		*insD*–*insC*	*insD, insC*
2406075	C→T	100%	V51I (GTT→ATT)	*nuoF* ←	NADH:ubiquinone oxidoreductase, chain F
2416102	G→A	100%	L233L (CTG→TTG)	*yfbS* ←	transporter
2538628	C→T	100%	T258T (ACC→ACT)	*cysK* →	cysteine synthase A, O-acetylserine sulfhydrolase A

position	mutation	freq	annotation	gene	description
					subunit
2865228	A→G	3.8%	L288P (CTG→CCG)	*rpoS* ←	RNA polymerase, sigma S (sigma 38) factor
2865274	+46 bp	76.7%	coding (817/876 nt)	*rpoS* ←	RNA polymerase, sigma S (sigma 38) factor
2865278	+92 bp	61.6%	coding (813/876 nt)	*rpoS* ←	RNA polymerase, sigma S (sigma 38) factor
2866111	A→G	100%	intergenic (-21/+159)	*rpoS* ← / ← *nlpD*	RNA polymerase, sigma S (sigma 38) factor/outer membrane lipoprotein
3003330	G→A	100%	L371L (CTC→CTT)	*ygeV* ←	DNA-binding transcriptional regulator
3049815	G→T	100%	R387S (CGC→AGC)	*visC* ←	oxidoreductase
3397813	C→G	100%	D141H (GAT→CAT)	*yhdE* ←	hypothetical protein
3449851	G→A	100%	A494V (GCG→GTG)	*rpoC* ←	RNA polymerase, beta prime subunit
3742936	Δ1,336 bp	100%		*insD*–*insC*	*insD, insC*
3746911	Δ1,199 bp	100%		*insH*	*insH*
3750122	Δ1 bp	96.8%	coding (365/1416 nt)	*tnaA* ←	tryptophanase/ L-cysteine desulfhydrase, PLP-dependent
3750123	C→T	3.2%	G122S (GGC→AGC)	*tnaA* ←	tryptophanase/ L-cysteine desulfhydrase, PLP-dependent
3750591	Δ1,199 bp	100%		*insH*	*insH*
3898657	C→G	100%	L259V	*yiaJ* →	DNA-binding

position	mutation	freq	annotation	gene	description
			(CTG→GTG)		transcriptional repressor
3958123	Δ34 bp	100%	coding (1156-1189/1287 nt)	*dctA* →	C4-dicarboxylic acid, orotate and citrate transporter
4090749	A→T	100%	Q135L (CAG→CTG)	*malQ* →	4-alpha-glucanotransferase
4165991	A→C	100%	K43T (AAA→ACA)	*rpsL* →	30S ribosomal protein S12
4310363	Δ1,199 bp	100%		*insH–[alsK]*	*insH, [alsK]*
4371271	Δ2 bp	100%	intergenic (-6/+299)	*dcuA* ← / ← *aspA*	C4-dicarboxylate antiporter/aspartate ammonia-lyase
4546535	T→A	100%	intergenic (+296/-182)	*fimB* → / → *fimE*	tyrosine recombinase/inversion of on/off regulator of fimA/tyrosine recombinase/inversion of on/off regulator of fimA

Table 3.2 (SCV, isolate 2)

position	mutation	freq	annotation	gene	description
66457	C→A	100%	V32F (GTT→TTT)	*AraD* ←	L-ribulose-5-phosphate 4-epimerase
97880	C→T	100%	S265F (TCC→TTC)	*murD* →	UDP-N-acetylmuramoyl-L-alanine:D-glutamate ligase
104633	A→C	1.8%	I218L (ATC→CTC)	*ftsA* →	ATP-binding cell division protein involved in recruitment of FtsK to Z ring
167632	C→A	100%	P50Q	*fhuA* →	ferrichrome

position	mutation	freq	annotation	gene	description
			(CCG→CAG)		outer membrane transporter
274547	A→G	100%	intergenic (-206/-2)	*insH* ← / → *mmuP*	IS5 transposase and trans-activator/ S-methylmethionine transporter
274733	Δ97,240 bp	100%		*[mmuP]*–*[mhpD]*	***90 genes [mmuP], mmuM, afuC, afuB, insB, insA, ykgN, yagB, yagA, yagE, yagF, yagG, yagH, yagI, argF, insB, insA, yagJ, yagK, yagL, yagM, yagN, intF, yagP, yagQ, yagR, yagS, yagT, yagU, ykgJ, yagV, yagW, yagX, yagY, yagZ, ykgK, ykgL, Y75_p4288, Y75_p4289, eaeH, insE, insF, ykgA, ykgB, ykgI, ykgC, ykgD, ykgE, ykgF, ykgG, ykgH, betA, betB, betI, betT, yahA, yahB, yahC, yahD, yahE, yahF, yahG, yahH, yahI, yahJ, yahK, yahL, yahM, yahN, yahO, prpR,***

position	mutation	freq	annotation	gene	description
					prpB, prpC, prpD, prpE, codB, codA, cynR, cynT, cynS, cynX, lacA, lacY, lacZ, lacI, mhpR, mhpA, mhpB, mhpC, [mhpD]
547694	A→G	100%	intergenic (+123/-1156)	*fdrA* → / → *ylbF*	acyl-CoA synthetase with NAD(P)-binding Rossmann-fold domain/hypothetical protein
547831	+G	100%	intergenic (+260/-1019)	*fdrA* → / → *ylbF*	acyl-CoA synthetase with NAD(P)-binding Rossmann-fold domain/hypothetical protein
556858	A→T	100%	L36Q (CTG→CAG)	*folD* ←	bifunctional 5,10-methylene--tetrahydrofolate dehydrogenase/ 5,10-methylene--tetrahydrofolate cyclohydrolase
569996	G→C	100%	intergenic (+345/-120)	*ybcK* → / → *ybcL*	recombinase/kinase inhibitor
654214	Δ1,199 bp	100%		*insH*	*insH*
749354	C→A	100%	P17P (CCG→CCT)	*ybgO* ←	fimbrial-like adhesin protein
758440	C→A	100%	F110L (TTC→TTA)	*sdhB* →	succinate dehydrogenase, FeS subunit
887234	C→A	100%	A176A (GCG→GCT)	*ybjJ* ←	transporter
987574	G→T	100%	intergenic	*ompF* ← / ←	outer

position	mutation	freq	annotation	gene	description
			(-170/+433)	*asnS*	membrane porin 1a (Ia;b;F)/asparaginyl tRNA synthetase
1093686	T→C	100%	V130A (GTA→GCA)	*ycdT* →	diguanylate cyclase
1097580	C→T	23.7%	intergenic (+329/+226)	*ycdU* → / ← *serX*	inner membrane protein/tRNA-Ser
1170573	C→T	100%	intergenic (-164/-77)	*ycfQ* ← / → *ycfR*	DNA-binding transcriptional regulator/hypothetical protein
1301041	Δ1,336 bp	100%		*insC*–*insD*	*insC, insD*
1318876	G→A	100%	P21S (CCT→TCT)	*trpA* ←	tryptophan synthase subunit alpha
1413639	Δ23,060 bp	100%		*[ydaO]*–*ynaE*	***30 genes [ydaO], intR, ydaQ, ydaC, lar, recT, recE, racC, ydaE, kil, sieB, ydaF, ydaG, racR, ydaS, ydaT, ydaU, ydaV, ydaW, rzpR, rzoR, trkG, ynaK, ydaY, ynaA, insH, stfR, tfaR, pinR, ynaE***
1483588	G→A	100%	L322L (CTG→CTA)	*ynbD* →	phosphatase, inner membrane protein
1496775	Δ1 bp	100%	coding (11/924 nt)	*ydcI* ←	DNA-binding transcriptional regulator
1615814	C→A	100%	D202Y (GAC→TAC)	*yneI* ←	aldehyde dehydrogenase
1669599	Δ1 bp	100%	coding	*dgsA* ←	DNA-binding

position	mutation	freq	annotation	gene	description
			(680/1221 nt)		transcriptional repressor
1844073	G→A	100%	intergenic (-224/-12)	*ynjH* ← / → *gdhA*	hypothetical protein/glutamate dehydrogenase, NADP-specific
1918872	T→A	100%	S301T (TCC→ACC)	*yebS* →	inner membrane protein
1973476	G→A	100%	L310L (CTC→CTT)	*tar* ←	methyl-accepting chemotaxis protein II
2005401	C→T	100%	E115K (GAA→AAA)	*fliC* ←	flagellar filament structural protein
2170094	C→A	100%	intergenic (-437/+32)	*ogrK* ← / ← *yegR*	DNA-binding transcriptional regulator/hypothetical protein
2178210	A→C	3.0%	intergenic (+179/+183)	*insH* → / ← *gatZ*	IS5 element protein/D-tagatose 1,6-bisphosphate aldolase 2, subunit
2320795	Δ1,336 bp	100%		*insD–insC*	*insD, insC*
2406075	C→T	100%	V51I (GTT→ATT)	*nuoF* ←	NADH:ubiquinone oxidoreductase, chain F
2416102	G→A	100%	L233L (CTG→TTG)	*yfbS* ←	transporter
2538628	C→T	100%	T258T (ACC→ACT)	*cysK* →	cysteine synthase A, O-acetylserine sulfhydrolase A subunit
2865228	A→G	2.5%	L288P (CTG→CCG)	*rpoS* ←	RNA polymerase, sigma S (sigma 38) factor

position	mutation	freq	annotation	gene	description
2865274	+46 bp	76.2%	coding (817/876 nt)	*rpoS* ←	RNA polymerase, sigma S (sigma 38) factor
2865278	+92 bp	57.6%	coding (813/876 nt)	*rpoS* ←	RNA polymerase, sigma S (sigma 38) factor
2866111	A→G	100%	intergenic (-21/+159)	*rpoS* ← / ← *nlpD*	RNA polymerase, sigma S (sigma 38) factor/outer membrane lipoprotein
3003330	G→A	100%	L371L (CTC→CTT)	*ygeV* ←	DNA-binding transcriptional regulator
3049815	G→T	100%	R387S (CGC→AGC)	*visC* ←	oxidoreductase
3397813	C→G	100%	D141H (GAT→CAT)	*yhdE* ←	hypothetical protein
3449851	G→A	100%	A494V (GCG→GTG)	*rpoC* ←	RNA polymerase, beta prime subunit
3742936	Δ1,336 bp	100%		*insD–insC*	*insD, insC*
3746911	Δ1,199 bp	100%		*insH*	*insH*
3750122	Δ1 bp	100%	coding (365/1416 nt)	*tnaA* ←	tryptophanase/ L-cysteine desulfhydrase, PLP-dependent
3750591	Δ1,199 bp	100%		*insH*	*insH*
3898657	C→G	100%	L259V (CTG→GTG)	*yiaJ* →	DNA-binding transcriptional repressor
3958123	Δ34 bp	100%	coding (1156-1189/1287 nt)	*dctA* →	C4-dicarboxylic acid, orotate and citrate transporter
4090749	A→T	100%	Q135L (CAG→CTG)	*malQ* →	4-alpha-glucanotransferase
4165991	A→C	100%	K43T	*rpsL* →	30S ribosomal

position	mutation	freq	annotation	gene	description
			(AAA→ACA)		protein S12
4310363	Δ1,199 bp	100%		*insH–[alsK]*	*insH, [alsK]*
4371271	Δ2 bp	100%	intergenic (-6/+299)	*dcuA* ← / ← *aspA*	C4-dicarboxylate antiporter/aspartate ammonia-lyase
4519481	T→C	4.6%	D626G (GAT→GGT)	*fecA* ←	ferric citrate outer membrane transporter
4546535	T→A	100%	intergenic (+296/-182)	*fimB* → / → *fimE*	tyrosine recombinase/inversion of on/off regulator of fimA/tyrosine recombinase/inversion of on/off regulator of fimA

Table 3.3 (LCV, isolate 1)

position	mutation	freq	annotation	gene	description
66457	C→A	100%	V32F (GTT→TTT)	*araD* ←	L-ribulose-5-phosphate 4-epimerase
97880	C→T	100%	S265F (TCC→TTC)	*murD* →	UDP-N-acetylmuramoyl-L-alanine:D-glutamate ligase
167632	C→A	100%	P50Q (CCG→CAG)	*fhuA* →	ferrichrome outer membrane transporter
274547	A→G	100%	intergenic (-206/-2)	*insH* ← / → *mmuP*	IS5 transposase and trans-activator/S-methylmethionine transporter
274733	Δ97,240 bp	100%		*[mmuP]–[mhpD]*	***90 genes [mmuP],***

					mmuM, afuC, afuB, insB, insA, ykgN, yagB, yagA, yagE, yagF, yagG, yagH, yagI, argF, insB, insA, yagJ, yagK, yagL, yagM, yagN, intF, yagP, yagQ, yagR, yagS, yagT, yagU, ykgJ, yagV, yagW, yagX, yagY, yagZ, ykgK, ykgL, Y75_p4288, Y75_p4289, eaeH, insE, insF, ykgA, ykgB, ykgI, ykgC, ykgD, ykgE, ykgF, ykgG, ykgH, betA, betB, betI, betT, yahA, yahB, yahC, yahD, yahE, yahF, yahG, yahH, yahI, yahJ, yahK, yahL, yahM, yahN, yahO, prpR, prpB, prpC, prpD, prpE, codB, codA, cynR, cynT, cynS, cynX, lacA, lacY, lacZ, lacI, mhpR, mhpA, mhpB, mhpC, [mhpD]
547694	A→G	100%	intergenic (+123/-1156)	*fdrA* → / → *ylbF*	acyl-CoA synthetase with NAD(P)-binding Rossmann-fold

					domain/hypothetical protein
547831	+G	100%	intergenic (+260/-1019)	*fdrA* → / → *ylbF*	acyl-CoA synthetase with NAD(P)-binding Rossmann-fold domain/hypothetical protein
556858	A→T	100%	L36Q (CTG→CAG)	*folD* ←	bifunctional 5,10-methylene-tetrahydrofolate dehydrogenase/5,10-methylene-tetrahydrofolate cyclohydrolase
569996	G→C	100%	intergenic (+345/-120)	*ybcK* → / → *ybcL*	recombinase/kinase inhibitor
654214	Δ1,199 bp	100%		*insH*	*insH*
749354	C→A	100%	P17P (CCG→CCT)	*ybgO* ←	fimbrial-like adhesin protein
758440	C→A	100%	F110L (TTC→TTA)	*sdhB* →	succinate dehydrogenase, FeS subunit
758562	T→A	100%	L151H (CTC→CAC)	*sdhB* →	succinate dehydrogenase, FeS subunit
887234	C→A	100%	A176A (GCG→GCT)	*ybjJ* ←	transporter
987574	G→T	100%	intergenic (-170/+433)	*ompF* ← / ← *asnS*	outer membrane porin 1a (Ia;b;F)/asparaginyl tRNA synthetase
1093686	T→C	100%	V130A (GTA→GCA)	*ycdT* →	diguanylate cyclase
1097580	C→T	19.1%	intergenic (+329/+226)	*ycdU* → / ← *serX*	inner membrane protein/tRNA-Ser
1170573	C→T	100%	intergenic (-164/-77)	*ycfQ* ← / → *ycfR*	DNA-binding transcriptional regulator/hypothetical protein

1197797	C→T	100%	H366H (CAC→CAT)	*icd* →	isocitrate dehydrogenase ,-specific for NADP+
1301041	Δ1,336 bp	100%		*insC–insD*	*insC, insD*
1318876	G→A	100%	P21S (CCT→TCT)	*trpA* ←	tryptophan synthase subunit alpha
1413639	Δ23,060 bp	100%		*[ydaO]–ynaE*	***30 genes [ydaO], intR, ydaQ, ydaC, lar, recT, recE, racC, ydaE, kil, sieB, ydaF, ydaG, racR, ydaS, ydaT, ydaU, ydaV, ydaW, rzpR, rzoR, trkG, ynaK, ydaY, ynaA, insH, stfR, tfaR, pinR, ynaE***
1483588	G→A	100%	L322L (CTG→CTA)	*ynbD* →	phosphatase, inner membrane protein
1496775	Δ1 bp	100%	coding (11/924 nt)	*ydcI* ←	DNA-binding transcriptional regulator
1555625	G→A	100%	intergenic (-73/+61)	*adhP* ← / ← *sfcA*	alcohol dehydrogenase , 1-propanol preferring/malate dehydrogenase , NAD-requiring
1615814	C→A	100%	D202Y (GAC→TAC)	*yneI* ←	aldehyde dehydrogenase
1669599	Δ1 bp	100%	coding (680/1221 nt)	*dgsA* ←	DNA-binding transcriptional repressor
1844073	G→A	100%	intergenic (-224/-12)	*ynjH* ← / → *gdhA*	hypothetical protein/glutamate dehydrogenase ,

					NADP-specific
1918872	T→A	100%	S301T (TCC→ACC)	*yebS* →	inner membrane protein
1973476	G→A	100%	L310L (CTC→CTT)	*tar* ←	methyl-accepting chemotaxis protein II
2005401	C→T	100%	E115K (GAA→AAA)	*fliC* ←	flagellar filament structural protein
2170094	C→A	100%	intergenic (-437/+32)	*ogrK* ← / ← *yegR*	DNA-binding transcriptional regulator/hypothetical protein
2320795	Δ1,336 bp	100%		*insD–insC*	*insD, insC*
2406075	C→T	100%	V51I (GTT→ATT)	*nuoF* ←	NADH:ubiquinone oxidoreductase, chain F
2416102	G→A	100%	L233L (CTG→TTG)	*yfbS* ←	transporter
2538628	C→T	100%	T258T (ACC→ACT)	*cysK* →	cysteine synthase A, O-acetylserine sulfhydrolase A subunit
2866111	A→G	100%	intergenic (-21/+159)	*rpoS* ← / ← *nlpD*	RNA polymerase, sigma S (sigma 38) factor/outer membrane lipoprotein
3003330	G→A	100%	L371L (CTC→CTT)	*ygeV* ←	DNA-binding transcriptional regulator
3049815	G→T	100%	R387S (CGC→AGC)	*visC* ←	oxidoreductase
3397813	C→G	100%	D141H (GAT→CAT)	*yhdE* ←	hypothetical protein
3406254	Δ3 bp	12.3%	coding (483-485/1350 nt)	*accC* →	acetyl-CoA carboxylase, biotin carboxylase subunit

3742936	Δ1,336 bp	100%		*insD–insC*	*insD, insC*
3746911	Δ1,199 bp	100%		*insH*	*insH*
3750122	Δ1 bp	95.0%	coding (365/1416 nt)	*tnaA* ←	tryptophanase/ L-cysteine desulfhydrase, PLP-dependent
3750123	C→T	4.6%	G122S (GGC→AGC)	*tnaA* ←	tryptophanase/ L-cysteine desulfhydrase, PLP-dependent
3898657	C→G	100%	L259V (CTG→GTG)	*yiaJ* →	DNA-binding transcriptional repressor
3958123	Δ34 bp	100%	coding (1156-1189/1287 nt)	*dctA* →	C4-dicarboxylic acid, orotate and citrate transporter
4090749	A→T	100%	Q135L (CAG→CTG)	*malQ* →	4-alpha-glucanotransferase
4165991	A→C	100%	K43T (AAA→ACA)	*rpsL* →	30S ribosomal protein S12
4310363	Δ1,199 bp	100%		*insH–[alsK]*	*insH, [alsK]*
4371271	Δ2 bp	100%	intergenic (-6/+299)	*dcuA* ← / ← *aspA*	C4-dicarboxylate antiporter/aspartate ammonia-lyase
4546535	T→A	100%	intergenic (+296/-182)	*fimB* → / → *fimE*	tyrosine recombinase/inversion of on/off regulator of fimA/tyrosine recombinase/inversion of on/off regulator of fimA

Table 3.4 (LCV, isolate2)

**position**	**mutation**	**freq**	**annotation**	*gene*	**description**
66457	C→A	100%	V32F (GTT→TTT)	*araD* ←	L-ribulose-5-phosphate 4-epimerase
97880	C→T	100%	S265F	*murD* →	UDP-N-acetyl

			(TCC→TTC)		muramoyl-L-alanine:D-glutamate ligase
167632	C→A	100%	P50Q (CCG→CAG)	*fhuA* →	ferrichrome outer membrane transporter
274547	A→G	100%	intergenic (-206/-2)	*insH* ← / → *mmuP*	IS5 transposase and trans-activator/ S-methylmethionine transporter
274733	Δ97,240 bp	100%		*[mmuP]*–*[mhpD]*	***90 genes [mmuP], mmuM, afuC, afuB, insB, insA, ykgN, yagB, yagA, yagE, yagF, yagG, yagH, yagI, argF, insB, insA, yagJ, yagK, yagL, yagM, yagN, intF, yagP, yagQ, yagR, yagS, yagT, yagU, ykgJ, yagV, yagW, yagX, yagY, yagZ, ykgK, ykgL, Y75_p4288, Y75_p4289, eaeH, insE, insF, ykgA, ykgB, ykgI, ykgC, ykgD, ykgE, ykgF, ykgG, ykgH, betA, betB, betI, betT, yahA, yahB, yahC, yahD, yahE, yahF, yahG, yahH, yahI, yahJ, yahK, yahL, yahM, yahN,***

					yahO, prpR, prpB, prpC, prpD, prpE, codB, codA, cynR, cynT, cynS, cynX, lacA, lacY, lacZ, lacI, mhpR, mhpA, mhpB, mhpC, [mhpD]
547694	A→G	100%	intergenic (+123/-1156)	*fdrA* → / → *ylbF*	acyl-CoA synthetase with NAD(P)-binding Rossmann-fold domain/hypothetical protein
547831	+G	100%	intergenic (+260/-1019)	*fdrA* → / → *ylbF*	acyl-CoA synthetase with NAD(P)-binding Rossmann-fold domain/hypothetical protein
556858	A→T	100%	L36Q (CTG→CAG)	*folD* ←	bifunctional 5,10-methylene-tetrahydrofolate dehydrogenase/5,10-methylene-tetrahydrofolate cyclohydrolase
569996	G→C	100%	intergenic (+345/-120)	*ybcK* → / → *ybcL*	recombinase/kinase inhibitor
749354	C→A	100%	P17P (CCG→CCT)	*ybgO* ←	fimbrial-like adhesin protein
758440	C→A	100%	F110L (TTC→TTA)	*sdhB* →	succinate dehydrogenase, FeS subunit
758562	T→A	100%	L151H (CTC→CAC)	*sdhB* →	succinate dehydrogenase, FeS subunit
887234	C→A	100%	A176A (GCG→GCT)	*ybjJ* ←	transporter
987574	G→T	100%	intergenic (-170/+433)	*ompF* ← / ← *asnS*	outer membrane

					porin 1a (Ia;b;F)/asparaginyl tRNA synthetase
1093686	T→C	100%	V130A (GTA→GCA)	*ycdT* →	diguanylate cyclase
1097580	C→T	18.8%	intergenic (+329/+226)	*ycdU* → / ← *serX*	inner membrane protein/tRNA-Ser
1170573	C→T	100%	intergenic (-164/-77)	*ycfQ* ← / → *ycfR*	DNA-binding transcriptional regulator/hypothetical protein
1197797	C→T	100%	H366H (CAC→CAT)	*icd* →	isocitrate dehydrogenase,-specific for NADP+
1301041	Δ1,336 bp	100%		*insC–insD*	*insC, insD*
1318876	G→A	100%	P21S (CCT→TCT)	*trpA* ←	tryptophan synthase subunit alpha
1413639	Δ23,060 bp	100%		*[ydaO]–ynaE*	***30 genes [ydaO], intR, ydaQ, ydaC, lar, recT, recE, racC, ydaE, kil, sieB, ydaF, ydaG, racR, ydaS, ydaT, ydaU, ydaV, ydaW, rzpR, rzoR, trkG, ynaK, ydaY, ynaA, insH, stfR, tfaR, pinR, ynaE***
1483588	G→A	100%	L322L (CTG→CTA)	*ynbD* →	phosphatase, inner membrane protein
1496775	Δ1 bp	100%	coding (11/924 nt)	*ydcI* ←	DNA-binding transcriptional regulator
1555625	G→A	100%	intergenic (-73/+61)	*adhP* ← / ← *sfcA*	alcohol dehydrogenase, 1-propanol

					preferring/malate dehydrogenase, NAD-requiring
1615814	C→A	100%	D202Y (GAC→TAC)	*yneI* ←	aldehyde dehydrogenase
1669599	Δ1 bp	100%	coding (680/1221 nt)	*dgsA* ←	DNA-binding transcriptional repressor
1844073	G→A	100%	intergenic (-224/-12)	*ynjH* ← / → *gdhA*	hypothetical protein/glutamate dehydrogenase, NADP-specific
1907281	C→A	1.9%	intergenic (+308/-121)	*yobD* → / → *yebN*	inner membrane protein/inner membrane protein
1918872	T→A	100%	S301T (TCC→ACC)	*yebS* →	inner membrane protein
1973476	G→A	100%	L310L (CTC→CTT)	*tar* ←	methyl-accepting chemotaxis protein II
2005401	C→T	100%	E115K (GAA→AAA)	*fliC* ←	flagellar filament structural protein
2170094	C→A	100%	intergenic (-437/+32)	*ogrK* ← / ← *yegR*	DNA-binding transcriptional regulator/hypothetical protein
2320795	Δ1,336 bp	100%		*insD–insC*	*insD, insC*
2406075	C→T	100%	V51I (GTT→ATT)	*nuoF* ←	NADH:ubiquinone oxidoreductase, chain F
2416102	G→A	100%	L233L (CTG→TTG)	*yfbS* ←	transporter
2538628	C→T	100%	T258T (ACC→ACT)	*cysK* →	cysteine synthase A, O-acetylserine sulfhydrolase A subunit

2866111	A→G	100%	intergenic (-21/+159)	*rpoS* ← / ← *nlpD*	RNA polymerase, sigma S (sigma 38) factor/outer membrane lipoprotein
3003330	G→A	100%	L371L (CTC→CTT)	*ygeV* ←	DNA-binding transcriptional regulator
3049815	G→T	100%	R387S (CGC→AGC)	*visC* ←	oxidoreductase
3397813	C→G	100%	D141H (GAT→CAT)	*yhdE* ←	hypothetical protein
3742936	Δ1,336 bp	100%		*insD–insC*	*insD, insC*
3750122	Δ1 bp	100%	coding (365/1416 nt)	*tnaA* ←	tryptophanase/ L-cysteine desulfhydrase, PLP-dependent
3750591	Δ1,199 bp	100%		*insH*	*insH*
3898657	C→G	100%	L259V (CTG→GTG)	*yiaJ* →	DNA-binding transcriptional repressor
3958123	Δ34 bp	100%	coding (1156-1189/1287 nt)	*dctA* →	C4-dicarboxylic acid, orotate and citrate transporter
4090749	A→T	100%	Q135L (CAG→CTG)	*malQ* →	4-alpha-glucanotransferase
4165991	A→C	100%	K43T (AAA→ACA)	*rpsL* →	30S ribosomal protein S12
4310363	Δ1,199 bp	100%		*insH–[alsK]*	*insH, [alsK]*
4353540	T→C	1.1%	intergenic (-118/+453)	*dcuB* ← / ← *dcuR*	C4-dicarboxylate antiporter/DNA-binding response regulator in two-component regulatory system with DcuS
4371271	Δ2 bp	100%	intergenic (-6/+299)	*dcuA* ← / ← *aspA*	C4-dicarboxylate antiporter/aspartate

					ammonia-lyase
4546535	T→A	100%	intergenic (+296/-182)	*fimB* → / → *fimE*	tyrosine recombinase/inversion of on/off regulator of fimA/tyrosine recombinase/inversion of on/off regulator of fimA

Table 3.5 (Mixed population)

position	mutation	freq	annotation	gene	description
66457	C→A	100%	V32F (GTT→TTT)	*araD* ←	L-ribulose-5-phosphate 4-epimerase
97880	C→T	100%	S265F (TCC→TTC)	*murD* →	UDP-N-acetylmuramoyl-L-alanine:D-glutamate ligase
167632	C→A	100%	P50Q (CCG→CAG)	*fhuA* →	ferrichrome outer membrane transporter
274547	A→G	100%	intergenic (-206/-2)	*insH* ← / → *mmuP*	IS5 transposase and trans-activator/S-methylmethionine transporter
274733	Δ97,240 bp	100%		*[mmuP]*–*[mhpD]*	***90 genes [mmuP], mmuM, afuC, afuB, insB, insA, ykgN, yagB, yagA, yagE, yagF, yagG, yagH, yagI, argF, insB, insA, yagJ, yagK, yagL, yagM, yagN, intF, yagP, yagQ, yagR, yagS,***

					yagT, yagU, ykgJ, yagV, yagW, yagX, yagY, yagZ, ykgK, ykgL, Y75_p4288, Y75_p4289, eaeH, insE, insF, ykgA, ykgB, ykgI, ykgC, ykgD, ykgE, ykgF, ykgG, ykgH, betA, betB, betI, betT, yahA, yahB, yahC, yahD, yahE, yahF, yahG, yahH, yahI, yahJ, yahK, yahL, yahM, yahN, yahO, prpR, prpB, prpC, prpD, prpE, codB, codA, cynR, cynT, cynS, cynX, lacA, lacY, lacZ, lacI, mhpR, mhpA, mhpB, mhpC, [mhpD]
547694	A→G	100%	intergenic (+123/-1156)	*fdrA* → / → *ylbF*	acyl-CoA synthetase with NAD(P)-binding Rossmann-fold domain/hypothetical protein
547831	+G	100%	intergenic (+260/-1019)	*fdrA* → / → *ylbF*	acyl-CoA synthetase with NAD(P)-binding Rossmann-fold domain/hypothetical protein
556858	A→T	100%	L36Q (CTG→CAG)	*folD* ←	bifunctional 5,10-methylene-tetrahydrofolate

					dehydrogenase /5,10-methylene-tetrahydrofolate cyclohydrolase
569996	G→C	100%	intergenic (+345/-120)	*ybcK* → / → *ybcL*	recombinase/kinase inhibitor
654214	Δ1,199 bp	100%		*insH*	*insH*
749354	C→A	100%	P17P (CCG→CCT)	*ybgO* ←	fimbrial-like adhesin protein
758440	C→A	44.7%	F110L (TTC→TTA)	*sdhB* →	succinate dehydrogenase, FeS subunit
758562	T→A	5.2%	L151H (CTC→CAC)	*sdhB* →	succinate dehydrogenase, FeS subunit
887234	C→A	100%	A176A (GCG→GCT)	*ybjJ* ←	transporter
987574	G→T	100%	intergenic (-170/+433)	*ompF* ← / ← *asnS*	outer membrane porin 1a (Ia;b;F)/asparaginyl tRNA synthetase
1093686	T→C	100%	V130A (GTA→GCA)	*ycdT* →	diguanylate cyclase
1097580	C→T	18.4%	intergenic (+329/+226)	*ycdU* → / ← *serX*	inner membrane protein/tRNA-Ser
1170573	C→T	100%	intergenic (-164/-77)	*ycfQ* ← / → *ycfR*	DNA-binding transcriptional regulator/hypothetical protein
1197797	C→T	3.2%	H366H (CAC→CAT)	*icd* →	isocitrate dehydrogenase,-specific for NADP+
1197809	C→T	2.7%	T370T (ACC→ACT)	*icd* →	isocitrate dehydrogenase,-specific for NADP+
1197822	T→C	2.6%	L375L (TTA→CTA)	*icd* →	isocitrate dehydrogenase,-specific for NADP+

1197824	A→G	2.6%	L375L (TTA→TTG)	*icd* →	isocitrate dehydrogenase ,-specific for NADP+
1197857	G→C	1.4%	A386A (GCG→GCC)	*icd* →	isocitrate dehydrogenase ,-specific for NADP+
1301041	Δ1,336 bp	100%		*insC–insD*	*insC, insD*
1306963	C→T	5.1%	P166S (CCG→TCG)	*oppD* →	oligopeptide transporter subunit
1318876	G→A	100%	P21S (CCT→TCT)	*trpA* ←	tryptophan synthase subunit alpha
1413639	Δ23,060 bp	100%		*[ydaO]–ynaE*	***30 genes [ydaO], intR, ydaQ, ydaC, lar, recT, recE, racC, ydaE, kil, sieB, ydaF, ydaG, racR, ydaS, ydaT, ydaU, ydaV, ydaW, rzpR, rzoR, trkG, ynaK, ydaY, ynaA, insH, stfR, tfaR, pinR, ynaE***
1483588	G→A	100%	L322L (CTG→CTA)	*ynbD* →	phosphatase, inner membrane protein
1496775	Δ1 bp	100%	coding (11/924 nt)	*ydcI* ←	DNA-binding transcriptional regulator
1555625	G→A	4.6%	intergenic (-73/+61)	*adhP* ← / ← *sfcA*	alcohol dehydrogenase , 1-propanol preferring/malate dehydrogenase , NAD-requiring
1615814	C→A	100%	D202Y (GAC→TAC)	*yneI* ←	aldehyde dehydrogenase

1669599	Δ1 bp	100%	coding (680/1221 nt)	*dgsA* ←	DNA-binding transcriptional repressor
1844073	G→A	100%	intergenic (-224/-12)	*ynjH* ← / → *gdhA*	hypothetical protein/glutamate dehydrogenase, NADP-specific
1918872	T→A	100%	S301T (TCC→ACC)	*yebS* →	inner membrane protein
1973476	G→A	100%	L310L (CTC→CTT)	*tar* ←	methyl-accepting chemotaxis protein II
2005401	C→T	100%	E115K (GAA→AAA)	*fliC* ←	flagellar filament structural protein
2170094	C→A	100%	intergenic (-437/+32)	*ogrK* ← / ← *yegR*	DNA-binding transcriptional regulator/hypothetical protein
2320795	Δ1,336 bp	100%		*insD–insC*	*insD, insC*
2406075	C→T	100%	V51I (GTT→ATT)	*nuoF* ←	NADH:ubiquinone oxidoreductase, chain F
2416102	G→A	100%	L233L (CTG→TTG)	*yfbS* ←	transporter
2538628	C→T	100%	T258T (ACC→ACT)	*cysK* →	cysteine synthase A, O-acetylserine sulfhydrolase A subunit
2733780	T→A	11.3%	E296D (GAA→GAT)	*rluD* ←	23S rRNA pseudouridine synthase
2865228	A→G	5.1%	L288P (CTG→CCG)	*rpoS* ←	RNA polymerase, sigma S (sigma 38) factor
2865274	+46 bp	21.5%	coding (817/876 nt)	*rpoS* ←	RNA polymerase, sigma S (sigma 38) factor

2865278	+92 bp	8.9%	coding (813/876 nt)	*rpoS* ←	RNA polymerase, sigma S (sigma 38) factor
2866111	A→G	100%	intergenic (-21/+159)	*rpoS* ← / ← *nlpD*	RNA polymerase, sigma S (sigma 38) factor/outer membrane lipoprotein
3003330	G→A	100%	L371L (CTC→CTT)	*ygeV* ←	DNA-binding transcriptional regulator
3049815	G→T	100%	R387S (CGC→AGC)	*visC* ←	oxidoreductase
3397813	C→G	100%	D141H (GAT→CAT)	*yhdE* ←	hypothetical protein
3449851	G→A	13.6%	A494V (GCG→GTG)	*rpoC* ←	RNA polymerase, beta prime subunit
3450056	C→A	22.3%	A426S (GCA→TCA)	*rpoC* ←	RNA polymerase, beta prime subunit
3742936	Δ1,336 bp	100%		*insD–insC*	*insD, insC*
3746911	Δ1,199 bp	100%		*insH*	*insH*
3750122	Δ1 bp	97.5%	coding (365/1416 nt)	*tnaA* ←	tryptophanase/ L-cysteine desulfhydrase, PLP-dependent
3750123	C→T	2.5%	G122S (GGC→AGC)	*tnaA* ←	tryptophanase/ L-cysteine desulfhydrase, PLP-dependent
3898657	C→G	100%	L259V (CTG→GTG)	*yiaJ* →	DNA-binding transcriptional repressor
3958123	Δ34 bp	99.6%	coding (1156-1189/1287 nt)	*dctA* →	C4-dicarboxylic acid, orotate and citrate transporter
4090749	A→T	100%	Q135L (CAG→CTG)	*malQ* →	4-alpha-glucanotransferase
4165991	A→C	100%	K43T	*rpsL* →	30S ribosomal

			(AAA→ACA)		protein S12
4200197	A→G	36.1%	K271E (AAA→GAA)	*rpoA* →	RNA polymerase subunit alpha
4310363	Δ1,199 bp	100%		*insH–[alsK]*	*insH, [alsK]*
4346527	C→G	3.9%	intergenic (-221/-62)	*melR* ← / → *melA*	DNA-binding transcriptional dual regulator/alpha--galactosidase
4346883	T→A	3.3%	F99I (TTC→ATC)	*melA* →	alpha-galactosi dase
4347339	C→T	2.8%	R251C (CGC→TGC)	*melA* →	alpha-galactosi dase
4371271	Δ2 bp	100%	intergenic (-6/+299)	*dcuA* ← / ← *aspA*	C4-dicarboxyla te antiporter/aspa rtate ammonia-lyase
4371461	T→C	2.4%	intergenic (-196/+110)	*dcuA* ← / ← *aspA*	C4-dicarboxyla te antiporter/aspa rtate ammonia-lyase
4546535	T→A	100%	intergenic (+296/-182)	*fimB* → / → *fimE*	tyrosine recombinase/in version of on/off regulator of fimA/tyrosine recombinase/in version of on/off regulator of fimA
